# Evaluation of splenic accumulation and colocalization of immature reticulocytes and *Plasmodium vivax* in asymptomatic malaria: A prospective human splenectomy study

**DOI:** 10.1371/journal.pmed.1003632

**Published:** 2021-05-26

**Authors:** Steven Kho, Labibah Qotrunnada, Leo Leonardo, Benediktus Andries, Putu A. I. Wardani, Aurelie Fricot, Benoit Henry, David Hardy, Nur I. Margyaningsih, Dwi Apriyanti, Agatha M. Puspitasari, Pak Prayoga, Leily Trianty, Enny Kenangalem, Fabrice Chretien, Valentine Brousse, Innocent Safeukui, Hernando A. del Portillo, Carmen Fernandez-Becerra, Elamaran Meibalan, Matthias Marti, Ric N. Price, Tonia Woodberry, Papa A. Ndour, Bruce M. Russell, Tsin W. Yeo, Gabriela Minigo, Rintis Noviyanti, Jeanne R. Poespoprodjo, Nurjati C. Siregar, Pierre A. Buffet, Nicholas M. Anstey

**Affiliations:** 1 Global and Tropical Health Division, Menzies School of Health Research and Charles Darwin University, Darwin, Northern Territory, Australia; 2 Eijkman Institute for Molecular Biology, Jakarta, Indonesia; 3 Timika Malaria Research Program, Papuan Health and Community Development Foundation, Timika, Papua, Indonesia; 4 Rumah Sakit Umum Daerah Kabupaten Mimika, Timika, Papua, Indonesia; 5 UMR_S1134, BIGR, Inserm, Université de F-75015 Paris, and Laboratory of Excellence GR-Ex, Paris, France; 6 Institut Pasteur, Experimental Neuropathology Unit, Paris, France; 7 Department of Biological Sciences, Notre Dame University, Notre Dame, Indiana, United States of America; 8 ISGlobal, Hospital Clinic-Universitat de Barcelona, Barcelona, Spain; 9 Germans Trias I Pujol Research Institute, Badalona, Spain; 10 Catalan Institution for Research and Advanced Studies, Barcelona, Spain; 11 Department of Immunology and Infectious Diseases, Harvard School of Public Health, Boston, Massachusetts, United States of America; 12 Center for Excellence in Vascular Biology, Department of Pathology, Brigham and Women’s Hospital, Boston, Massachusetts, United States of America; 13 Wellcome Center for Integrative Parasitology, University of Glasgow, Glasgow, United Kingdom; 14 Center for Tropical Medicine and Global Health, Nuffield Department of Medicine, University of Oxford, Oxford, United Kingdom; 15 Mahidol-Oxford Tropical Medicine Research Unit, Faculty of Tropical Medicine, Mahidol University, Bangkok, Thailand; 16 Department of Microbiology and Immunology, University of Otago, Dunedin, New Zealand; 17 Department of Pediatrics, University of Gadjah Mada, Yogyakarta, Indonesia; 18 Department of Anatomical Pathology, Rumah Sakit Cipto Mangunkusumo and Universitas Indonesia, Jakarta, Indonesia; Mahidol-Oxford Tropical Medicine Research Unit, THAILAND

## Abstract

**Background:**

A very large biomass of intact asexual-stage malaria parasites accumulates in the spleen of asymptomatic human individuals infected with *Plasmodium vivax*. The mechanisms underlying this intense tropism are not clear. We hypothesised that immature reticulocytes, in which *P*. *vivax* develops, may display high densities in the spleen, thereby providing a niche for parasite survival.

**Methods and findings:**

We examined spleen tissue in 22 mostly untreated individuals naturally exposed to *P*. *vivax* and *Plasmodium falciparum* undergoing splenectomy for any clinical indication in malaria-endemic Papua, Indonesia (2015 to 2017). Infection, parasite and immature reticulocyte density, and splenic distribution were analysed by optical microscopy, flow cytometry, and molecular assays. Nine non-endemic control spleens from individuals undergoing spleno-pancreatectomy in France (2017 to 2020) were also examined for reticulocyte densities. There were no exclusion criteria or sample size considerations in both patient cohorts for this demanding approach.

In Indonesia, 95.5% (21/22) of splenectomy patients had asymptomatic splenic *Plasmodium* infection (7 *P*. *vivax*, 13 *P*. *falciparum*, and 1 mixed infection). Significant splenic accumulation of immature CD71 intermediate- and high-expressing reticulocytes was seen, with concentrations 11 times greater than in peripheral blood. Accordingly, in France, reticulocyte concentrations in the splenic effluent were higher than in peripheral blood. Greater rigidity of reticulocytes in splenic than in peripheral blood, and their higher densities in splenic cords both suggest a mechanical retention process. Asexual-stage *P*. *vivax*-infected erythrocytes of all developmental stages accumulated in the spleen, with non-phagocytosed parasite densities 3,590 times (IQR: 2,600 to 4,130) higher than in circulating blood, and median total splenic parasite loads 81 (IQR: 14 to 205) times greater, accounting for 98.7% (IQR: 95.1% to 98.9%) of the estimated total-body *P*. *vivax* biomass. More reticulocytes were in contact with sinus lumen endothelial cells in *P*. *vivax*- than in *P*. *falciparum*-infected spleens. Histological analyses revealed 96% of *P*. *vivax* rings/trophozoites and 46% of schizonts colocalised with 92% of immature reticulocytes in the cords and sinus lumens of the red pulp. Larger splenic cohort studies and similar investigations in untreated symptomatic malaria are warranted.

**Conclusions:**

Immature CD71^+^ reticulocytes and splenic *P*. *vivax-*infected erythrocytes of all asexual stages accumulate in the same splenic compartments, suggesting the existence of a cryptic endosplenic lifecycle in chronic *P*. *vivax* infection. Findings provide insight into *P*. *vivax*-specific adaptions that have evolved to maximise survival and replication in the spleen.

## Abstract 2

Dr. Anstey and co-authors found that P. vivax-infected immature reticulocytes and erythrocytes accumulate in the same splenic compartments, suggesting existence of a cryptic endosplenic lifecycle in chronic P. vivax infection that maximizes survival and replication in the spleen.

## Author summary

**Why was this study done?:**

- There is a surprisingly large biomass of intact *Plasmodium vivax* parasites in the spleen of people living in malaria endemic areas, greater than with *Plasmodium falciparum*.
- Why *P*. *vivax* accumulates so intensely in the spleen is not known.
- *P*. *vivax* requires very young red cells (immature reticulocytes) for invasion and replication.

**What did the researchers do and find?:**

- The accumulation of *P*. *vivax* and immature reticulocytes were evaluated in spleens from people undergoing splenectomy in malaria-endemic Papua.
- *P*. *vivax* parasites and immature reticulocytes accumulated in the same splenic compartments.
- All stages of *P*. *vivax* accumulated in the spleen at magnitudes unexplainable by replication occurring in the circulation alone, with the proportion of each stage in the spleen consistent with the duration in their lifecycle.

**What do these findings mean?:**

- Taking advantage of the large physiological splenic reservoir of immature reticulocytes, the majority of the *P*. *vivax* lifecycle can take place in the spleen in chronic malaria.
- Chronic vivax malaria should be considered predominantly an infection of the reticulocyte-rich spleen, with secondary involvement of the intravascular compartment.
- The spleen is not solely a compartment for parasite destruction and clearance.
- Specific adaptations have likely evolved to maximise *P*. *vivax* survival in the spleen.

## Introduction

Malaria, mainly due to *Plasmodium falciparum* (Pf) and *Plasmodium vivax* (Pv), affects 228 million people with over 400,000 deaths each year [1]. The multistep life cycle of malaria parasites in humans involves 2 cell types: hepatocytes and erythrocytes. Initial hepatic tropism is clinically silent, while subsequent parasite development in erythrocytes almost invariably causes symptoms in nonimmune individuals and is thought to restrict parasite spread in the human body almost exclusively to the intravascular compartment.

Despite a growing body of research in Pv over the last 2 decades [2,3], vivax malaria remains less understood and presents a greater challenge to elimination than its more prevalent and virulent Pf counterpart [4]. A key biological feature of Pv not present in Pf is its ability to cause relapse from dormant stages in the liver. Hospitalisation with severe vivax malaria has been widely reported [5–11] and can lead to death [12,13], despite Pv displaying lower circulating parasite densities than Pf [14,15]. Lower parasitaemia has been attributed to the strict tropism of Pv for reticulocytes [16], a population of erythrocyte precursors originating in the bone marrow and found at low levels in peripheral blood. Such low parasitaemias have long been considered to indicate a low total parasite biomass in vivax malaria. However, computational Pv modelling based on *Plasmodium cynomolgi* growth data [17] and indirect measures in clinical Pv [18] point to a hidden subpopulation of parasites related to disease severity and residing in non-endothelial lined compartments [15]. Several reports in clinical Pv have confirmed the presence of intact asexual and sexual-stage infected erythrocytes (IEs) in the bone marrow [19–23] and spleen [23–25].

The spleen eliminates malaria parasites after antimalarial treatment [26–28] and has been assumed to be primarily an organ for parasite destruction. However, our recent evaluation of individuals undergoing splenectomy in malaria-endemic Papua, Indonesia, has found a very large biomass of intact asexual-stage Pv and Pf-IEs hidden in the spleen in chronic asymptomatic infections [29]. Splenic tropism was greater in Pv than Pf [29], but the reason for this is unclear. Direct evidence to support a cryptic Pv lifecycle in this organ remains lacking. Immature reticulocytes, characterised by expression of the CD71 transferrin receptor [30], are preferentially invaded by Pv for replication [31,32]. Animal studies [33–37] and a single early report in humans [38] have described reticulocyte pooling in the spleen. Whether the human spleen is concentrated with the immature reticulocyte subpopulations preferentially invaded by Pv has not been investigated.

Here, we extend our findings from the initial cohort with additional patients and experiments to characterise splenic reticulocyte subpopulations, *Plasmodium* developmental stages, and their co-compartmentalization in the human spleen, hypothesising the existence of an endosplenic asexual lifecycle in chronic asymptomatic Pv.

## Methods

### Splenectomy patients and samples

#### Setting

Rumah-Sakit-Umum-Daerah (RSUD) is the major regional hospital in the Mimika District of Papua, Indonesia. In this region, malaria transmission is unstable, with a blood parasitaemia prevalence of 14% and 38% by microscopy and polymerase chain reaction (PCR), respectively, and with approximately equal proportions of Pf and Pv infection [39].

#### Enrolment

The research team was on-call 24 hours a day for any patients undergoing elective splenectomy or explorative laparotomy for potential spleen rupture at RSUD hospital. All consenting patients undergoing splenectomy for any clinical indication were enrolled in the study between 2015 and 2017, as previously reported in brief [29]. There were no exclusion criteria or sample size considerations for this demanding approach. Demographic and clinical data were collected, including automated blood counts.

#### Management

As described previously in brief [29], a locally feasible protocol for the management of splenectomised patients was implemented based on international guidelines [40]. Upon recovery, patients were given post-splenectomy meningococcal, pneumococcal, *Haemophilus* and influenza vaccinations with Menveo, Prevenar 13, Hiberix, and Influvac. Local antimalarial treatment consisting of 3 days dihydroartemisinin-piperaquine, plus 14 days primaquine for *P*. *vivax* infections [41], was given to patients found to have *Plasmodium* infection by blood smear microscopy. Before discharge, patients were tested for human immunodeficiency virus and provided with standby antibiotics (3 g amoxicillin) in case of fever. Local clinicians and the District Health Authority deemed daily antibiotic prophylaxis unsustainable. Malaria radical cure for all patients undergoing splenectomy was introduced at the end of the study upon analysis of malaria risk [42].

### Sample collection

#### Day of surgery

The decision to proceed to splenectomy was made by the treating surgeon on clinical grounds. Eighteen millilitres of peripheral blood was collected intraoperatively into 3 anticoagulants: ethylenediaminetetraacetic acid (EDTA), lithium-heparin, and acid citrate dextrose (ACD) (BD Biosciences, Australia). Any remaining blood collected preoperatively that was to be discarded was also collected. All spleen tissue removed at the time of surgery (intact or in portions) was collected by the research team in the operating theatre. Histopathology is not routine at RSUD. Any splenectomy patients that were transfused prior to surgery had a sample of donor blood taken from the transfusion bag. Thick and thin blood smears were prepared from each blood sample and stained with 3% Giemsa solution (Merck, Darmstadt, Germany) for 45 minutes at room temperature (RT).

#### Spleen dissection

The spleen was immediately transferred to the hospital laboratory. Blood on the surface of the spleen was removed. Images and spleen weight were recorded (**S1A Fig**). Using sterile equipment and in a clean work area, the spleen was sliced approximately in half as shown in **S1B Fig** and one half set aside to generate formalin-fixed paraffin-embedded blocks (FFPE). The remaining half was sliced longitudinally into multiple sections of 0.5 to 1 cm thickness (**S1C Fig**). Sliced-spleen blood was collected as detailed below. Damaged tissue was avoided in all sample processing. Blood and spleen samples were transferred at RT to the research laboratory for further experiments.

#### Sliced-spleen blood collection

Tissue sections of approximately 1 cm^3^ in size were sampled randomly from spleen slices. Three pieces were added to each of up to eighteen 50 mL falcon tubes containing 15 mL of sterile RPMI 1640 medium (Thermofisher, Massachusetts, United States) supplemented with either EDTA, heparin or ACD (Sigma Aldrich, Missouri, US). Suspensions were incubated at RT on a slow rotator, or on the bench and inverting every 5 minutes, for up to 90 minutes. Spleen pieces were removed and the suspensions pooled for each anticoagulant through 70 μm SmartStrainers (Miltenyi Biotec, Bergisch Gladbach, Germany), then centrifuged at 500*g* for 15 minutes. Supernatants were discarded and the pellets resuspended in equal volumes of RPMI 1640 medium. The resulting suspensions were termed sliced-spleen blood with EDTA, heparin, or ACD. Thick and thin blood smears were prepared for sliced-spleen blood and stained with 3% Giemsa solution for 45 minutes at RT.

#### Biopsy and blood for molecular assays

Up to fifteen 0.5 cm punch biopsies were collected randomly from spleen slices and snap frozen in liquid nitrogen. EDTA-anticoagulated peripheral and sliced-spleen blood samples were centrifuged at 600*g* for 10 minutes. Aliquots of the resulting packed red blood cell (RBC) pellets were stored at −80°C.

#### Formalin-fixed paraffin-embedded blocks

The unused spleen half was sliced longitudinally to 0.5 to 1 cm in thickness and fixed at RT for 48 to 72 hours in 1 L of 10% neutral-buffered formalin (NBF; containing 100 mL 37% to 40% formaldehyde, 900 mL deionised water, 6.5 g Na_2_HPO_4_, and 4 g NaH_2_PO_4_). NBF was replaced every 24 hours. After fixation, 0.5 cm punch biopsies were taken randomly from spleen slices and placed into histology cassettes. To dehydrate the tissue, fixed biopsies were incubated in absolute ethanol for 20 minutes at 60°C, then placed in fresh ethanol and incubated again until a total of four 20-minute incubations were performed. Fixed biopsies were then incubated in pure xylene for 10 minutes at RT, drained, then submerged in Paraplast Plus (Leica Biosystems, Wetzlar, Germany) for 30 minutes at 60°C. Fixed biopsies were carefully positioned in metal base moulds, filled with Paraplast Plus, covered with labelled cassettes, and incubated for 15 minutes at −20°C to solidify. FFPE were detached from the base moulds and stored at RT until further use.

#### Total immunoglobulin M (IgM)

In patients with massively large spleens, total IgM levels were measured to confirm or infer the suspected diagnosis of Hyperreactive Malarial Splenomegaly (HMS). Frozen plasma from lithium-heparin peripheral blood was used for total IgM measurement using a BN ProSpec protein analyser (Siemens Healthineers, Erlangen, Germany). Frozen plasma from 54 adults collected in a household survey [39] were used as controls representing baseline IgM levels in the Timika population.

#### Follow-up

Peripheral blood was collected again from splenectomised patients ≥2 months after splenectomy and processed identically. Patients were given meningococcal booster vaccinations with Menveo and undertook clinical checkup by a research clinician. Bimonthly interviews were conducted for up to 14 months to collect data on post-splenectomy malaria or other illnesses. Reported malaria episodes were confirmed with the treating hospital or health clinic.

### *Plasmodium* detection and quantification

#### Rapid Diagnostic Test (RDT) and blood smear microscopy

Peripheral blood was tested for *Plasmodium* detection using the First Response Malaria Ag (*Plasmodium* lactate dehydrogenase [pLDH]/histidine-rich protein-2 [HRP2]) Combo RDT as per manufacturer’s instructions (Premier Medical Corp., Daman, India). Giemsa-stained slides of peripheral blood and sliced-spleen blood were examined by 2 expert microscopists for the presence of *Plasmodium*-infected erythrocytes as previously described [29] using CX31 LED microscopes (Olympus, Tokyo, Japan). At least 40 thick smear high-power fields (HPFs) were examined at 1,000× magnification. Peripheral blood parasitaemia and parasite staging was determined in the thick smear as parasites per 500 white blood cells (WBCs). Automated WBC counts were used to calculate the number of parasites per μL peripheral blood. Non-phagocytosed peripheral IEs as a percentage of total circulating RBCs was also calculated for comparison to non-phagocytosed IEs in the spleen. In the comparisons of peripheral and splenic non-phagocytosed parasitaemias, individuals with submicroscopic PCR-positive peripheral parasitaemia were conservatively assigned a parasite count of 10 parasites per μL based on the lower limit of detection by an experienced microscopist (10 to 20 parasites per μL) [43], with PCR-negative peripheral parasitaemia assigned a value of zero per μL.

**Giemsa histopathology** was performed as described previously [29] and summarised below. FFPE were sliced to 5 μm in thickness and placed on poly-L-treated microscope slides. Slides were deparaffinised in xylene (3 × 5 minutes), then rehydrated in a series of alcohol baths; absolute ethanol (2 × 5 minutes), 90% ethanol (5 minutes), 70% ethanol (5 minutes), and water (10 minutes). Slides were stained with 10% Giemsa in water for 30 to 45 minutes at RT. After rinsing, slides were further stained with 0.5% aqueous acetic acid for 10 to 30 seconds, then rapidly washed in a series of alcohol baths (70%, 90%, and 100% ethanol). Slides were dipped in 2 xylene baths, followed by incubation in a third clean xylene bath for 10 minutes at RT. Coverslips were mounted on to stained slides with Entellan (Merck, Darmstadt, Germany).

Sections were analysed by an expert microscopist to quantify non-phagocytosed IEs using an Olympus CX31 microscope. A second expert microscopist validated parasite quantitation in 30% of patients, and a third external microscopist qualitatively reviewed 20% of the panel (**S1D Fig**). Architectural regions were distinguished into the red-pulp sinus lumen and cords, and white-pulp perifollicular zones (PFZ) and non-circulatory spaces. Ten HPFs were examined in each of the red and white-pulp/PFZ areas at 1,000× magnification. The area covered by red and white-pulp was quantified using a digital tracing tool to enable normalisation of HPF counts and estimations of total splenic biomass. Non-phagocytosed IE and uninfected RBCs were counted in each region, and parasites categorised into groups based on their developmental stages (rings/trophozoites, schizonts, gametocytes, or unclassifiable stages). Parasites in Giemsa stains of this type of tissue appear different to their traditional colouration and morphology in normal blood smears. Non-phagocytosed IEs were identified as red/pink RBCs containing at least 1 circular brown dot (parasite nucleus) with or without light blue parasite cytoplasm visible as previously validated [44]. The need to assess various Z-planes by fine focus was an important step to confirm non-phagocytosed IEs.

#### Calculations

The following calculations were performed, as previously reported [29]:

- IEs per μL peripheral blood
  $$=\frac{\left[IE\;count\;per\;500\;WBC]\times [WBC\;count\;per\;\mu L\;from\;analyser\right]}{500}$$
- Non-phagocytosed peripheral IEs as % of peripheral RBCs
  $$=\frac{IEs\;per\;\mu L\;peripheral\;blood}{RBC\;count\;per\;\mu L\;from\;analyser}\times 100$$
  Individuals with submicroscopic peripheral parasitaemia were conservatively assigned a parasite count value of 10 parasites per μL based on the lower limit of detection by an experienced microscopist (10 to 20 parasites per μL) [43].
- Non-phagocytosed splenic IEs as % of splenic RBCs
  $$=\frac{non‐phagocytosed\;IE\;count}{\left[non‐phagocytosed\;IE\;count]+[RBC\;count\right]}\times 100$$
  Note: IE and RBC counts were determined in each spleen zone, then normalised according to the proportion of each zone within a given area.
- Non-phagocytosed spleen-to-peripheral parasite density ratio
  $$=\frac{non‐phagocytosed\;splenic\;IEs\;as\;\%\;of\;splenic\;RBCs}{non‐phagocytosed\;peripheral\;IEs\;as\;\%\;peripheral\;RBCs}$$
  Note: Individuals that were PCR-negative in peripheral blood were assigned a peripheral parasite count of zero and therefore were not included in this ratio.
- Distribution of parasite developmental stages in the spleen
  (e.g., for schizonts)
  $$=\frac{non‐phagocytosed\;schizont\;IE\;count}{\left[total\;non‐phagocytosed\;IE\;count\right]}\times 1,000$$
  Note: IE counts were determined in each spleen zone, then normalised according to the proportion of each zone within a given area.
- Total non-phagocytosed parasite biomass in peripheral blood
  $$=[IEs\;per\;\mu L\;peripheral\;blood]\times [total\;peripheral\;blood\;volume,see\;below]\times 10^{6}$$
- Total peripheral blood volume in litres (Nadler’s method)
  $$male=[0.3669\times {\left[height\;in\;meters\right]}^{3}]+[0.03219\times [patient\;weight\;in\;kg]]+0.6041$$
  $$female=[0.3561\times {\left[height\;in\;meters\right]}^{3}]+[0.03308\times [patient\;weight\;in\;kg]]+0.1833$$
  where the average height of males is 1.58 meters and females is 1.47 meters in Indonesia [45]. Patient weights were available for a subset of individuals. Those that were missing were given a value corresponding to the national average of 59.5 kg for males and 54.8 kg for females [45].
- Total non-phagocytosed parasite biomass in the spleen
  $$=\frac{\left[IE\;count\;in\;HPFs]\times [spleen\;weight\right]}{\left[spleen\;volume\;per\;HPF\;in\;{cm}^{3},see\;below]\times [number\;of\;HPFs]\times [density\;of\;human\;spleen\;in\;g/{cm}^{3}\right]}$$
  where density of human spleen = 1.04 g/cm^3^, from [46].
  Note: Biomass was normalised according to the proportion of each zone within a given area.
- Spleen volume (in cm^3^) per HPF
  $$=\frac{\left[area\;of\;HPF\;in\;{mm}^{2}]\times [section\;depth\;in\;mm\right]}{1,000}$$
  Where area of HPF is πr^2^ (r = 100 μm on Olympus CX31 at 1,000× magnification), and section depth was 0.005 mm.
- Non-phagocytosed IE biomass in the spleen as a percentage of the total intravascular, bone marrow, and intrasplenic biomass
  $$=\frac{total\;non‐phagocytosed\;IE\;biomass\;in\;the\;spleen}{\left[total\;non‐phagocytosed\;IE\;biomass\;in\;the\;spleen,bone\;marrow,and\;peripheral\;blood\right]}\times 100$$
  Note: Total bone marrow Pv biomass is an estimation based on 2 assumptions. Firstly, we assumed a bone marrow Pv parasitaemia equal to peripheral blood and consisting of 80% asexual stages, as observed in a previous study comparing bone marrow aspirates and peripheral blood from patients with uncomplicated vivax malaria [20]. Secondly, in a 60-kg person, the bone marrow can be estimated to contain 1.9 × 10^11^ reticulocytes [47]. Assuming that the bone marrow releases 2.2 × 10^11^ RBCs per day [48], we conservatively estimate that at steady-state, there may be up to 4 times this amount present in the bone marrow consisting of normocytes and young to mature reticulocytes (given commonly reported maturation times between 1 and 3 days [31,49]), as well as any physiological retention.
  $$Bone\;marrow\;biomass=\frac{non‐phagocytosed\;peripheral\;IEs\;\%\;peripheral\;RBCs\;}{100}\times 4\times 2.2\times 10^{11}\times 0.8$$
- Non-phagocytosed spleen-to-peripheral biomass ratio
  $$=\frac{non‐phagocytosed\;IE\;biomass\;in\;the\;spleen}{non‐phagocytosed\;IE\;biomass\;in\;peripheral\;blood}$$
- Non-phagocytosed IEs in each spleen compartment as % of total non-phagocytosed IEs
  $$=\frac{\begin{array}{c} non‐phagocytosed\;IE\;count\;in\;cords,\\ sinus\;lumen,PFZ,or\;non‐circulatory\;whte\;pulp\;spaces \end{array}}{total\;non‐phagocytosed\;IE\;count}\times 100$$
  Note: IE counts were determined in each spleen zone, then normalised according to the proportion of each zone within a given area. The same calculation was performed for each parasite stage.
- Non-phagocytosed parasitaemia for each stage
  (e.g., for schizonts)
  $$=\frac{non‐phagocytosed\;schizont\;IE\;count}{\left[non‐phagocytosed\;IE\;count]+[RBC\;count\right]}\times 1,000$$

#### Pv-AMA1 and CD68 immunohistochemistry (IHC)

Rehydrated FFPE spleen sections were stained manually with antibodies against Pv apical membrane antigen-1 (PvAMA1) peptide, a marker for Pv merozoites/mature stages, as described previously [29,50]. A dilution of 1:200 was used, and with secondary detection using N-Histofine simple stain MAX PO (Nichirei Biosciences, Tokyo, Japan). For CD68 IHC, rehydrated spleen sections were stained with a monoclonal mouse anti-human CD68 (clone KP-1) antibody (Roche, Basel, Switzerland) followed by secondary detection with N-Histofine simple stain MAX PO using an automated Leica BOND-III platform (Leica Biosystems, Wetzlar, Germany). All sections were counterstained with hematoxylin.

#### Transmission electron microscopy

Glutaraldehyde (2.5%) in cacodylate buffer was used to fix spleen tissue, then postfixed with 2% osmium tetroxide and 2.5% potassium ferricyanide in distilled water. After dehydration in ethanol and infiltration with propylene oxide, tissue was embedded in spurr resin followed by a vacuum step. Staining of ultrathin 7-nm sections with 2% uranyl acetate and triple lead citrate was performed, then examined using a JEOL JEM-1010 electron microscope (Tokyo, Japan) at 80 kv. Images were captured on special negative films, developed, and scanned.

#### Ex vivo spleen perfusion

A mixed culture of Pf-IEs (FUP/CB strain) prepared accordingly [51] was perfused through an uninfected control human spleen for 2 hours under physiological flow conditions as published [28,44]. Upon completion, the spleen was fixed by gently perfusing 70 to 100 mL of 4% formaldehyde and then cut into blocks for further fixation. FFPE and Giemsa-stained spleen sections were prepared as described for Indonesian spleen samples.

#### DNA extraction

QIAamp DNA mini kits (QIAGEN, Hidlen, Germany) were used for extraction of genomic DNA (gDNA) from 2 snap frozen spleen tissue biopsies (8 to 25 mg per biopsy), peripheral packed RBCs, and sliced-spleen packed RBCs (both approximately 100 μL) per patient. Exact weights/volumes were recorded for each sample. DNA extraction of peripheral blood samples was performed according to the manufacturer’s spin protocol, except that the final elution was with 100 μL of buffer AE (supplied).

gDNA from spleen biopsies and sliced-spleen blood samples were extracted according to a modified version of the manufacturer’s tissue purification protocol. Briefly, spleen samples were incubated at 4°C overnight in 150 μL of ATL buffer (supplied). An additional 100 μL of ATL buffer was added and biopsies cut into smaller pieces. Proteinase K (supplied) was used to disrupt the tissues (30 μL for biopsies and 20 μL for sliced-spleen blood samples) on a slow shaker at 56°C for 3 hours. Samples were mixed with 200 μL of ethanol, vortexed, then applied to the QIAamp Mini spin columns (supplied). The remaining steps were performed according to the manufacturer’s instructions and the gDNA eluted as per peripheral blood samples. Extracted gDNA was stored at −20°C until further use.

#### Nested PCR

*Plasmodium* species confirmation and detection of submicroscopic infections was undertaken in duplicate in 2 separate laboratories using a nested PCR protocol described elsewhere [52,53]. One microliter gDNA template was used in the first nest, and 1 μL of the resulting product ran neat and/or diluted 1:20 in water for the second nest. Small subunit ribosomal RNA DNA clones of Pf, Pv, *Plasmodium malariae*, and *Plasmodium ovale* (MRA-177, MRA-178, MRA-179, and MRA-180; ATCC, Manassas, Virginia) were used as positive controls. The limit of detection of the assay was 0.2 parasites/μL [54]. Samples were considered positive for *Plasmodium* species if results were positive in both laboratories. If results were discrepant, samples were considered positive if a *Plasmodium* species was detected using a third confirmatory assay (real-time PCR). The spleen was considered positive if any of the spleen biopsies and/or sliced-spleen blood samples were positive for *Plasmodium* species.

#### Real-time PCR

A real-time PCR assay was developed for the detection Pf and Pv parasite DNA in gDNA samples using the QuantStudio-6 Flex Real-Time PCR System (Thermofisher, Massachusetts, US). Specific primer-probe pairs coupled to different fluorescent reporter dyes enabled multiple target products to be distinguished in a single reaction. Two microliters of gDNA template were added either neat or diluted up to 200-fold and run on 384-well plates in quadruplicate. Pf and Pv assays were run separately using previously published primers and probes [55] at a final primer-probe concentration of 0.8 μM and 0.4 μM, respectively. Human beta-actin primers and probe were included in each assay (both at a final concentration of 0.1 μM) as an endogenous target for quality control [56,57]. TaqMan Gene Expression Master Mix was added as a source of polymerase and other real-time PCR reaction components (Thermofisher, Massachusetts, US). Cycling parameters comprised 50°C for 2 minutes, 95°C for 10 minutes, and 50×; 95°C for 15 seconds, followed by 58°C for 1 minute for Pf or 60°C for 1 minute for Pv. The mean cycle threshold value was used if samples had >1 replicate positive. Those with a cycle threshold standard deviation >0.8 between replicates were repeated, or the outlier excluded if >2 replicates were positive.

### Reticulocyte assays

#### Samples

Two patient cohorts were included in the reticulocyte evaluations. In the Indonesian splenectomy cohort, peripheral blood, sliced-spleen blood, and paraffinised spleen tissue blocks were used for reticulocyte evaluations. Reticulocyte results from the Indonesian infected spleens provided impetus to examine a second cohort of uninfected control spleens in Paris, France.

In the second cohort, hospital patients undergoing spleno-pancreatectomy for any reason were enrolled between December 2017 and December 2020 as part of ongoing splenic physiology studies in Paris, an area non-endemic for malaria. Patients received normal medical and surgical care according to hospital protocols. Clinical data were collected including age, sex, and surgical information. Spleens were collected on the day of surgery according to published protocols [28]. The splenic artery was cannulated within 90 minutes of the surgical procedure. Spleens were macroscopically and microscopically normal. Spleens were flushed with 100 to 500 mL of Krebs-albumin solution (25 mmol NaHCO_3_, 118 mmol NaCl, 4.7 mmol KCl, 1.2 mmol MgSO_4_7H2O, 1.2 mmol NaH_2_PO_4_, CaCl_2_, 7 mmol glucose, and 5 gr Albumax-II, in 1 L of water) using a 3-mm catheter inserted into the artery and ligated tightly to allow flushing at physiological pressure, with several wash fractions collected consecutively from the vein into 5-mL lithium-heparin blood tubes or 50-mL polyethylene tubes. Transfusion-related perfusion experiments were conducted thereafter as reported [58].

#### Magnetic enrichment

Heparin-anticoagulated peripheral blood and sliced-spleen blood from Indonesian patients were magnetically enriched for reticulocytes within 24 hours of surgery. First, blood samples were centrifuged and had plasma/supernatant removed, then RBC pellets were resuspended in equal volumes of cold MACS buffer (1:20 ratio of MACS bovine-serum-albumin stock and autoMACS Rinsing Solution). Twelve μL of anti-CD71 MicroBeads were added per mL of reconstituted blood, mixed, then incubated for 15 minutes at 4°C. Labelled blood samples were centrifuged, supernatants discarded, and pellets washed and resuspended in equal volumes of cold MACS buffer. Labelled samples were kept cold until further use. A small volume was aliquoted for CD71 phenotyping by flow cytometry. MS Columns connected to 30 μm Pre-Separation Filters were attached to a magnetic octoMACS Separator. Columns were hydrated with cold MACS buffer, then carefully loaded with 2 mL of labelled sample per column. Cells labelled with anti-CD71 MicroBeads were retained in the columns, while non-labelled cells collected into individual falcon tubes. Once sample flow stopped, 1 mL of cold MACS buffer was added to wash each filter/column. Wash buffer was allowed to completely elute, then pre-separation filters were removed and columns were washed again twice with cold MACS buffer. To collect retained cells, columns were removed from the magnet and placed onto new falcon tubes. Cells were eluted by adding 1 mL of cold MACS buffer and the columns drained manually with supplied plungers. The multiple enriched suspensions were pooled into 2 tubes labelled as peripheral blood or sliced-spleen blood, then the total number of cells in each tube counted using a Neubauer chamber. All reagents and consumables were purchased from Miltenyi Biotec (Bergisch Gladbach, Germany).

#### Flow cytometry

In the Indonesian cohort, pre-enriched (5 μL) and enriched (200,000 cells) suspensions from peripheral blood and sliced-spleen blood samples were stained with a 3-colour flow cytometry panel consisting of anti-CD45 (clone HI30) conjugated to Alexa Fluor 488, anti-CD71 (clone CY1G4) conjugated to phycoerythrin, and the fluorescent nucleic acid dye SYTO61. Isotype controls comprised anti-IgG2a conjugated to phycoerythrin instead of anti-CD71. Stains were incubated for 20 minutes at RT in the dark, then at least 150,000 events acquired on a BD Accuri C6 flow cytometer with CFlow Sampler software (BD Biosciences, Australia). All antibodies were purchased from BioLegend (San Diego, California) and SYTO61 from Thermofisher (Massachusetts, US).

In the French cohort, spleen fractions were centrifuged and 2 μL of each pellet resuspended in 1 mL of 1% Albumax-II/phosphate-buffered saline (PBS) (Thermofisher, Massachusetts, US). Suspensions were stained with anti-CD45 (clone HI30) conjugated to phycoerythrin-cyanine-7 and anti-CD71 (clone CY1G4) conjugated to allophycocyanin (both from BioLegend, San Diego, California) for 20 minutes at 4°C. Samples were washed, then resuspended in diluted BD Retic-Count (thiazole orange) and incubated in the dark for 1 hour at RT. At least 150,000 events were acquired on a BD Accuri C6 flow cytometer with CFlow Sampler software.

#### CD71 IHC

FFPE were sliced to 5 μm in thickness and layered onto treated microscope slides. Sections underwent deparaffinisation, rehydration, and immunohistochemical staining using an automated Leica BOND-III platform (Leica Biosystems, Wetzlar, Germany) with a rabbit polyclonal anti-CD71 transferrin receptor primary antibody (Abcam, Cambridge, United Kingdom), followed by secondary detection with N-Histofine simple stain MAX PO (Nichirei Biosciences, Tokyo, Japan) and a hematoxylin counterstain. A conservative estimate of the number of CD71^+^ immature reticulocytes was determined by a research microscopist in the different spleen zones, presented as a percentage of RBCs (calculations were made as per parasitaemia calculations). CD71^+^ immature reticulocytes in the sinus lumen were categorised into non-adherent cells and those that appeared to be adhering to endothelial cells lining the sinus lumen. To confirm their appearance in the spleen sections, reticulocyte-enriched blood from a healthy donor was pelleted, fixed, embedded in paraffin, and stained with CD71, all following identical protocols for preparation of spleen tissue. Immature reticulocytes were identified as CD71^+^ cells of variable size without large nuclear staining and with refractory properties similar to RBCs. A minimum of 1,000 RBCs in the red-pulp zones and 150 RBCs in the white-pulp zones were counted for each spleen section.

#### Deformability

An in vitro assay for the measurement of reticulocyte deformability was performed on a subset of ACD-anticoagulated peripheral blood and sliced-spleen blood from Indonesian patients within 24 hours of surgery. WBC depletion was performed prior to microsphiltration using Plasmodipur filters as per manufacturer’s instructions (EuroProxima, Arnhem, the Netherlands). Microsphere tips that mimicked mechanical filtration in the spleen were prepared in Australia as previously described [59], dried overnight in a vacuum compartment, then transported at RT to Papua. Tips were stored at RT in sealed desiccated bags and rehydrated with 1% Albumax-II/PBS for experiments. Prior to commencing the study, dehydration and rehydration of tips were quality tested as described below and showed no difference in results compared to freshly prepared tips.

Fresh human RBCs were collected on the day of experiments for quality control of microsphere tips. RBCs were washed with PBS and resuspended at 1% haematocrit in 1% Albumax-II/PBS. Heated RBCs were prepared by incubating at 50°C for 20 minutes. Heated and normal RBCs were labelled with PKH-26 or PKH-67 Fluorescent Cell Linker kit for general cell membrane labelling (Sigma Aldrich, Missouri, US) as described elsewhere [60]. PKH-labelled heated and normal RBCs were added to WBC-free patient RBC samples, each making up 5% of total RBCs. The resulting RBC suspensions were adjusted to 2% haematocrit using 1% Albumax-II/PBS and termed as splenic or peripheral upstream samples.

Microsphere tips were washed by perfusing with 2 mL of flow solution (1% Albumax-II/PBS). A total of 600 μL of upstream sample was introduced upstream of the microsphere tip and perfused with 6 mL of flow solution through the microbead layer. A constant flow rate of 60 mL/hour was maintained using an electric syringe pump. Each sample was run in triplicate and the downstream samples retrieved into falcon tubes.

Quality control of microsphere tips was examined by comparing the retention rate of heated and normal RBCs within the microbead layer. Labelling of heated RBCs with PKH-26 and normal RBCs with PKH-67 allowed these populations to be distinguished by fluorescence (**S2A Fig**). Fifty microlitres of each upstream and downstream sample were diluted with 450 μL of flow solution and ran on a BD Accuri C6 flow cytometer with CFlow Sampler software (BD Biosciences, Australia). Three hundred thousand events were collected per sample. RBC retention rate was calculated using the following formula:

$$RBC\;retention\;rate=\frac{\left[\%upstream\;gate]-[\%downstream\;gate\right]}{\left[\%upstream\;gate\right]}\times 100$$

Microsphere tips with a mean retention rate of >95% for heated RBCs and <5% for normal RBCs were considered acceptable (**S2B Fig**).

Thin blood smears were prepared for each of the upstream and downstream samples and stained with the New Methylene Blue Reticulocyte Stain (Sigma Aldrich, Missouri, US) as per manufacturer’s instructions. Reticulocytes were counted by a research microscopist within 30 minutes of staining and expressed as a percentage of 3,000 RBCs. Reticulocyte retention rate was calculated using the following formula and presented as the mean of 3 replicates:

$$Retic\;\mathrm{retention}\;\mathrm{rate}=\frac{\left[\%upstream\;retic\;count]-[\%downstream\;retic\;count\right]}{\left[\%upstream\;retic\;count\right]}\times 100$$

### Parasite viability

Once established that non-phagocytosed IEs accumulated in the spleen (see Results), we determined by subculture whether IEs retrieved from the spleen tissue were viable.

#### Preparation and culture

The viability of *Plasmodium* IEs in the spleen and peripheral blood was examined by testing their ability to grow ex vivo using previously described culture methods [61,62]. Heparin-anticoagulated sliced-spleen blood and peripheral blood were used for culture on the day of splenectomy. WBCs were removed from samples prior to culture using Plasmodipur filters as per manufacturer’s instructions (EuroProxima, Arnhem, the Netherlands). Each WBC-free sample was cultured in 2 separate flasks containing different culture medium and at 1% haematocrit. One flask contained Pf culture media and comprised RPMI 1640 medium supplemented with L-glutamine (2 mM), HEPES (25 mM), NaHCO_3_ (20 mM), gentamicin (40 mg/L), D-glucose (0.25%), and human serum (10%). The second flask contained Pv culture media and comprised McCoy’s 5A medium (Thermofisher, Massachusetts, US) supplemented with L-glutamine (2 mM), HEPES (25 mM), NaHCO_3_ (20 mM), gentamicin (40 mg/L), D-glucose (0.25%), and human serum (20%). Flasks were supplemented with fresh human RBCs if peripheral or splenic RBCs from patients were insufficient for 1% haematocrit culture conditions. Human serum and fresh RBCs were obtained from nonexposed donors with matching blood group. Flasks were incubated in a candle jar at 37°C for up to 3 weeks. Culture media was replaced every 48 hours, and fresh human RBCs were added if parasitaemia exceeded 3%.

#### Monitoring by microscopy

Thick and thin blood smears were made for each flask on the day of splenectomy, and again every 24 hours to monitor parasite growth. Slides were stained daily with 3% Giemsa solution for 45 minutes at RT, then read by a research microscopist for the presence of *Plasmodium* IEs using an Olympus CX31 microscope. Parasites were counted in the thin smear per 5,000 RBCs, and the frequency of asexual stages (rings, trophozoites, schizonts) were determined.

### Statistics

Analyses and imaging were performed using GraphPad Prism 8 (GraphPad, California), ZEN 2 (Carl Zeiss, Germany) and FlowJo 10 (BD, Ashland). Minor adjustments to brightness/contrast were made to the entirety of some images which do not modify any original features. The Wilcoxon matched-pairs signed rank test was applied to paired datasets. The Mann–Whitney was used for between-group comparison on continuous variables and the chi-squared test for categorical variables. Correlations were analysed using Spearman tests (spleen weights were log-transformed). Two-sided *p*-values <0.05 were considered statistically significant.

### Ethical approval

The studies were approved by the Human Research Ethics Committees of Gadjah Mada University, Eijkman Institute for Molecular Biology, Indonesia, Menzies School of Health Research, Australia, and/or Ile-de-France II, France. In Indonesia, written informed consent was obtained from the patient’s relatives during surgery and from each patient postoperatively. Patient consent in the French cohort was obtained by the surgical team.

## Results

### High prevalence of *Plasmodium* infection in splenectomised patients

Twenty-two patients undergoing mostly trauma-related splenectomy were enrolled from 2015 to 2017 at RSUD Hospital in Timika (**Table 1**) which includes the addition of 7 patients to our first reported cohort [29]. The median age was 24.5 (range:12 to 46) years; 68.2% (15/22) were male, and 68.2% (15/22) of Papuan ethnicity. Two patients underwent elective splenectomy, 20 were splenectomised following trauma. Five patients (22.7%) were treated for malaria in the month before splenectomy, three within 3 days. There were no differences in parameters assessed between those who did or did not receive prior antimalarial treatment, therefore all patients were pooled for analysis. At splenectomy, patients were afebrile (≤37.5°C) and free of other malaria-related symptoms. Splenomegaly was present in all but 3 patients. Macroscopic features of spleens are shown in **S3 Fig**. The median spleen weight was 431*g* (range 80 to 1,918). Two patients with massive splenomegaly (patients #9 and #20) had total plasma IgM concentrations 2 standard deviations (SD) greater than the population mean (327 [SD = 385] mg/dL, *n =* 54 Timika matched-controls tested in parallel), a diagnostic criterion for HMS [63].

**Table 1:** Patient records and *Plasmodium* detection.

Age, Sex^a^, Ethnicity^b^	Reason for splenectomy	Body Temp (°C)	Spleen weight (grams)^c^	Total plasma IgM (mg/dL)	*Plasmodium* detection at surgery						Time to commencement of prior treatment	Malaria treatment after surgery^h^	First detected *Plasmodium* infection in the following 12 months			Patient ID^i^
					Peripheral RDT	Microscopy^d^			PCR							
						Peripheral blood	Spleen blood	Spleen histology	Peripheral blood	Spleen			Month	Microscopy	PCR	
32, M, NP	trauma	36.8	228 (N)	108	neg	neg	neg	neg	neg	neg	-	UT	-	-	-	19*
22, F, H	trauma	36.4	490 (S)	N/A	HRP2^+^Pan^+^	neg	neg	neg	neg	Pf	3 days (DHP+PQ)^f^	DHP+PQ	-	-	-	12
39, M, NP	trauma	36.4	142 (N)	<37.0	neg	neg	neg	Pf	neg	Pf ^e^	-	UT	-	-	-	11^#^
41, F, H	splenomegaly	36.3	1,918 (SS)	4200	neg	neg	neg	Pf	neg	Pf ^e^	-	UT	1	Pv^J^	N/A	20^#^^^^
16, M, H	trauma	N/A	690 (S)	320	neg	neg	Pf (a)	US	Pf	Pf	-	IV-ART, DHP	11	Pv^J^	N/A	3^^^
41, M, H	trauma	36.8	785 (S)	1,200	neg	neg	Pf (a)	Pf	Pf	Pf	-	T	12	Pf^J^	N/A	9^^^
20, M, L	trauma	37.3	761 (S)	265	neg	Pf (a)	Pf (a)	US	Pf	Pf	9.5 hours (IV-ART)	IV-ART	N/A	N/A	N/A	8
19, F, H	trauma	36.6	424 (S)	318	neg	Pf (a)	Pf (a)	US	Pf	Pf	-	T	2	neg	Mix Pf Pv	13
12, F, L	trauma	36.0	704 (S)	N/A	neg	Pf (a)	Pf (a)	Pf	N/A	Pf	-	T	N/A	N/A	N/A	14^^^
20, M, H	trauma	36.3	658 (S)	269	HRP2^+^Pan^+^	Pf (a,g)	Pf (a,g)	Pf	Pf	Pf	<1 month (DHP+PQ)^g^	DHP+PQ	2–4	Pv^J^	N/A	7^^^
15, M, L	trauma	36.2	335 (S)	190	HRP2^+^Pan^+^	Pf (a,g)	Pf (a,g)	Pf	Pf	Pf	3 days (DHP+PQ)^f^	DHP+PQ	2	Pf^J^	N/A	5^^^
30, M, NP	trauma	36.8	438 (S)	164	HRP2^+^Pan^+^	Pf (a)	Pf (a)	Pf	Pf	Pf	-	IV-ART, DHP+PQ	8	Pv^J^	N/A	10^^^
15, M, H	trauma	36.6	358 (S)	282	HRP2^+^Pan^+^	Pf (a,g)	Pf (a,g)	Pf	Pf	Pf	-	T	2	neg	Pf	17*^^^
40, M, NP	trauma	36.0	263 (S)	360	HRP2^+^Pan^+^	Pf (a,g)	Pf (a,g)	Pf	Pf	Pf	-	T	1	Pv^J^	N/A	15^^^
28, M, L	trauma	36.2	454 (S)	616	neg	neg	neg	N/A	Mix Pf Pv	Mix Pf Pv	-	T	2–3	Pv^J^	N/A	21
16, F, H	splenomegaly	36.9	1,250 (SS)	221	neg	neg	neg	Pv	Pv	Pv	-	T	1	Pv^J^	N/A	16^^^
46, M, L	trauma	37.0	279 (S)	72.4	neg	neg	neg	Pv	Pv	Pv	-	DHP+PQ^f^	1	Pv^J^	N/A	4*^^^
25, F, NP	trauma	36.9	80 (N)	<37.0	N/A	neg	neg	Pv	Pv	Pv	-	UT	N/A	N/A	N/A	22^^^
19, M, NP	trauma	36.0	130 (N)	<37.0	neg	neg	neg	Pv	Pv	Pv	-	UT	N/A	N/A	N/A	18*^^^
24, F, NP	trauma	36.5	300 (S)	96.0	neg	neg	Pv (a)	Pv	Pm	Pv	-	DHP+PQ	11	Pv^J^	N/A	2^^^
35, M, H	trauma	36.0	211 (N)	<37.0	neg	neg	Pv (a)	US	Pv	Pv	<1 month (DHP+PQ)^g^	IV-ART, DHP+PQ^f^	2	neg	Pv	6
36, M, H	trauma	36.5	446 (S)	153	neg	Pv (a)	Pv (a,g)	Pv	Pv	Pv	-	IV-ART	1	Pv^J^	N/A	1^^^

^a^M, male; F, female.

^b^H, highland Papuan; L, lowland Papuan; NP, non-Papuan.

^c^N, normal (<250 g); S, splenomegaly (250–1,000 g); SS, severe splenomegaly (>1,000 g).

^d^Parasites in blood smears were staged into: a, asexual stages; g, gametocytes.

^e^Positive by histology and by real-time PCR only.

^f^1-day PQ dose (Pf).

^g^14-day PQ dose (Pv or mix).

^h^DHP, dihydroartemisinin-piperaquine; IV-ART, intravenous artesunate; PQ, primaquine; T, unknown treatment; UT, untreated.

^i^Refer to the following patient IDs when referred to in text and figures.

^j^Symptomatic malaria.

Total plasma IgM concentrations 2 standard deviations (SD) greater than the population mean (327 [SD = 385] mg/dL, *n =* 54 Timika matched-controls tested in parallel), a diagnostic criterion for hyperreactive malarial splenomegaly.

There were no differences in parameters assessed between those who did or did not receive prior antimalarial treatment, therefore all patients were pooled for analysis.

Some patients received transfusion (*<60 minutes; ^#^>60 minutes) prior to sample collection. Transfused bloods were *Plasmodium*-negative by microscopy.

^^^Patients in previously published cohort [29].

None of the patients were seropositive for human immunodeficiency virus, and none had fever (≥37.5°C) or other malaria symptoms at surgery.

Refer to S4 Table for patient-automated blood counts.

Patients 18 and 22 died within 1 week after splenectomy due to multiple traumatic injuries. Patient 8 was lost to follow-up.

Missing data–patient 3 body temperature (not recorded); patient 12 IgM data (heparin blood not available); patient 14 IgM and PCR data (peripheral blood not available–peripheral RDT and slide results from hospital); patient 22 RDT result (not performed); PCR result at first recurrence (not tested for those with first recurrence identified from health facility records).

IgM, immunoglobulin M; N/A, not available (missing); Pf, *P*. *falciparum*; Pm, *P*. *malariae*; Pv, *P*. *vivax*; RDT, Rapid Diagnostic Test; US, unreadable slide.

**Fig 1:**
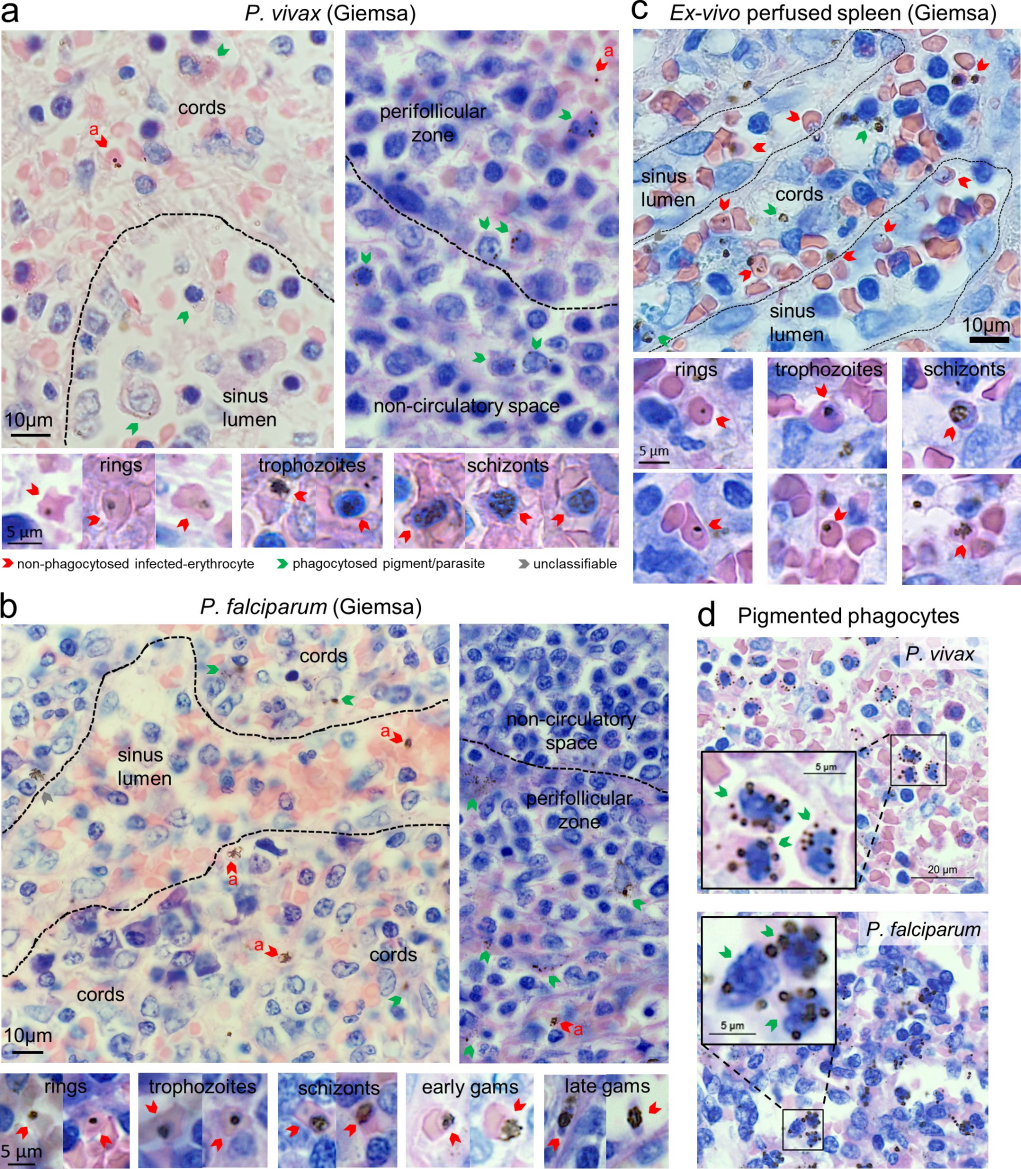
Counting malaria parasites on Giemsa-stained spleen sections of human spleens. Tissue sections stained with Giemsa were analysed in *P*. *vivax*- and *P*. *falciparum*-infected spleens at 400× magnification using a Carl Zeiss AxioScan Z1. Representative *P*. *vivax*-infected spleen sections are from patient #1 and *P*. *falciparum* from patient #17, with staging key of non-phagocytosed *P*. *vivax* and *P*. *falciparum* shown below these panels (a, asexuals) (a, b). Tissue compartments were categorised into white-pulp non-circulatory spaces and perifollicular zones, and red-pulp sinus lumen and cords. An uninfected control human spleen was perfused with *P*. *falciparum* lab strain cultures and had Giemsa-stained sections examined to validate the appearance of asexual-stage *Plasmodium* (c). Phagocytosed parasites (a–c) and pigmented phagocytes (d) were observed in Giemsa-stained spleen sections from infected individuals with representative images shown for each species.

In total, splenic *Plasmodium* infection was found in 21 of 22 (95.5%) splenectomised patients (**Table 2**; **Fig 1**). Seven were infected with Pv, 13 with Pf, and 1 with a mixed Pf-Pv infection. Spleen infection was more prevalent than blood infection, with peripheral parasitaemia positive by microscopy and/or PCR in 9 (40.9%) and 18 (81.8%), respectively. No individual had parasitaemia in peripheral blood but not in the spleen. One Pv and 2 Pf infections in the spleen were undetectable by PCR in peripheral blood, as reported [29]. Of the 20 patients surviving hospitalisation, 18 could be followed up post-discharge: 15 (83.3%) had at least 1 episode of recurrent parasitaemia within 12 months, 13 (72.2%) with symptomatic malaria (10 [55.5%] Pv; **Table 1**), in line with the increased risk of malaria following splenectomy in Papua, Indonesia, greater for Pv than Pf [42].

**Table 2:** Baseline characteristics of *n =* 22 splenectomy patients in Papua, Indonesia.

Parameters	All splenectomy patients *n* = 22	Subgroups				*P. vivax vs *P*. *falciparum* subgroup comparison *p*-value
		Asymptomatic *P*. *vivax n* = 7	Asymptomatic *P*. *falciparum n* = 13	Asymptomatic mixed *P*. *vivax-P*. *falciparum n* = 1	Uninfected control *n* = 1	
Age in years (median [IQR])	24.5 (18.3–36.8)	25 (19–36)	20 (15.5–39.5)	28	32	0.47
Sex (n/N of males, [%])	15/22 (68.2)	4/7 (57.1)	9/13 (69.2)	1 (100)	1 (100)	0.65
Ethnicity (n/N of Papuans, [%])	15/22 (68.2)	4/7 (57.1)	10/13 (76.9)	1 (100)	0	0.61
Splenectomy due to trauma (n/N, %)	20/22 (90.9)	6/7 (85.7)	12/13 (92.3)	1 (100)	1 (100)	0.65
Splenectomy due to splenomegaly (n/N, %)	2/22 (9.1)	1/7 (14.3)	1/13 (7.7)	0	0	1
Body temp. in °C (median [IQR])	36.5 (36.2–36.8)	36.5 (36–36.9)	36.4 (36.2–36.8)	36.2	36.8	0.57
Peripheral RDT (n/N of positive [%])	6/21 (28.6)	0	6/13 (46.2)	0	0	0.052
Peripheral microscopy (n/N of positive [%])	9/22 (40.9)	1/7 (14.3)	8/13 (61.5)	0	0	0.070
Spleen blood microscopy (n/N of positive [%])	13/22 (59.1)	3/7 (42.9)	10/13 (76.9)	0	0	0.17
Spleen histology (n/N of positive [%])	15/17 (88.2)	6/6 (100)	9/10 (90)	-	0	0.44
Peripheral blood PCR (n/N of positive [%])	17/21 (81.0)	7/7 (100)	9/12 (75)	1 (100)	0	0.16
Spleen PCR (n/N of positive [%])	21/22 (95.5)	7/7 (100)	13/13 (100)	1(100)	0	-
Treated with antimalarials in the last month (n/N, [%])	4/22 (18.2)	1/7 (14.3)	4/13 (30.8)	0	0	0.61
Treated with antimalarials after surgery (n/N, [%])	17/22 (77.3)	5/7 (71.4)	11/13 (84.6)	1 (100)	0	0.49
First infection in the next 12 months with *P*. *vivax* (n/N, [%])	11/18 (61.1)	5/5 (100)	5/11 (45.5)	1 (100)	0	**0.043**
First infection in the next 12 months with *P*. *falciparum* (n/N, [%])	3/18 (16.7)	0	3/11 (27.3)	0	0	0.51

IQR, interquartile range; RDT, rapid diagnostic test.

*Continuous variables were compared using the Mann–Whitney test, and categorical variables using the chi-squared test or Fisher exact test. *P* value <0.05 considered statistically significant.

In summary, we uncovered an overall prevalence of splenic *Plasmodium* infection of 95.5% in patients undergoing (mostly post-trauma) splenectomy who are living in a malaria-endemic area and with no malaria-related symptoms except for splenomegaly.

### Intense splenic tropism of intact non-phagocytosed asexual IEs in the spleen, greater in Pv than Pf

#### Validations

**Fig 2:**
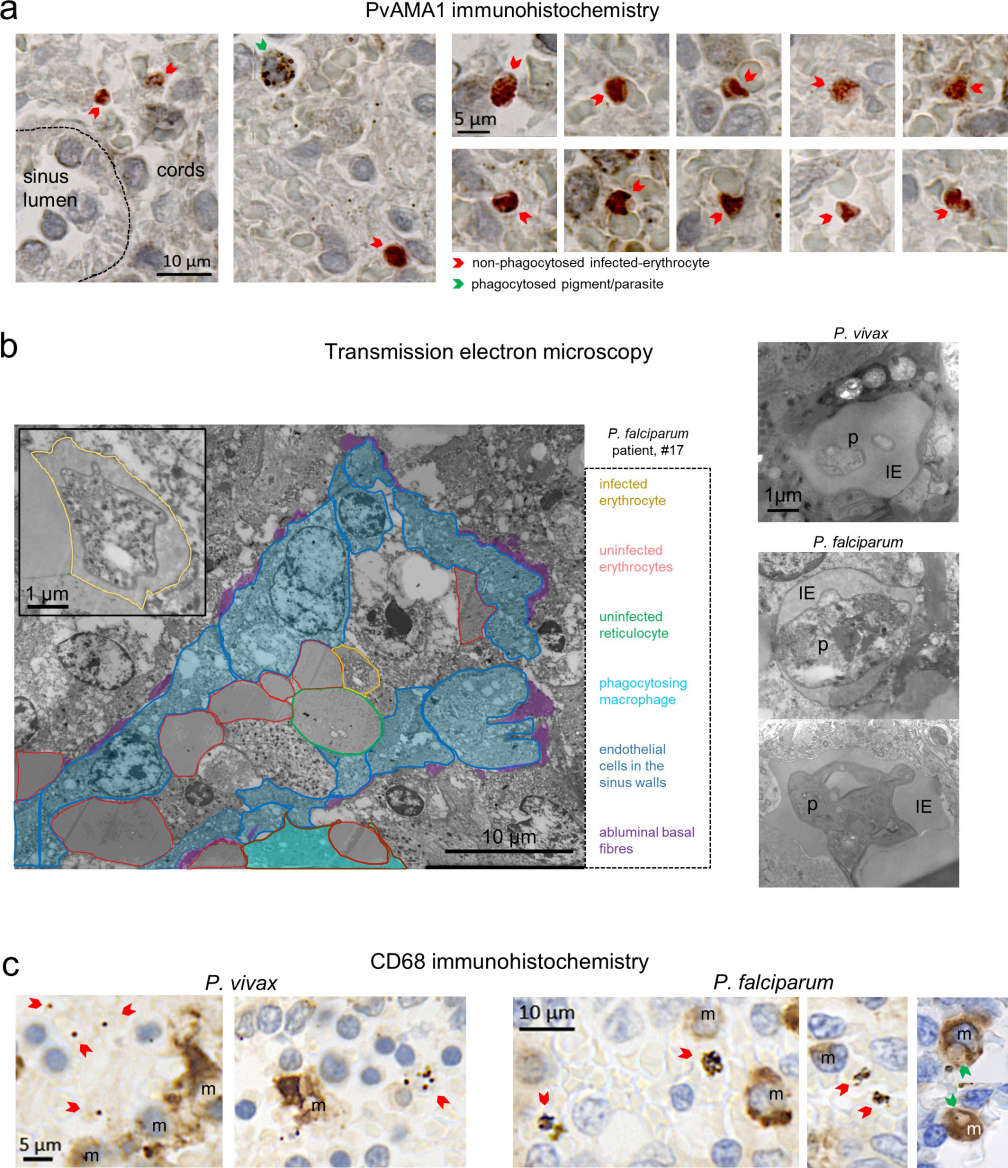
Confirming the integrity of non-phagocytosed IEs by immunohistochemistry and electron microscopy. Antibodies against PvAMA1, a marker for *P*. *vivax* merozoites/mature stages staining red, were tested on spleen sections from patient #1 (a) and others (S4D Fig) to confirm previous findings [29] on the presence of mature *P*. *vivax* stages. Spleen tissue from patients in the cohort was also examined by transmission electron microscopy to identify non-phagocytosed malaria parasites (p) inside erythrocytes (i), with representative images in both *P*. *vivax* (patient #2) and *P*. *falciparum* (patients #5 and #17) (b). IEs in the spleen were largely localised outside of macrophages (m) as illustrated in *P*. *vivax*- (patient #18) and *P*. *falciparum*-infected (patient #17) spleen sections stained with macrophage marker CD68, including examples of phagocytosed IEs shown on the right (c). IE, infected erythrocyte; m, macrophages; p, parasites; PvAMA1, Pv apical membrane antigen-1.

Non-phagocytosed IEs were validated as previously reported [29]. In brief, IEs were quantified in peripheral blood and spleens on Giemsa-stained smears and tissue sections, respectively (**Fig 1A and 1B, S4A and S4B Fig**). In addition, an uninfected non-trauma spleen in France perfused ex vivo with a mixture of Pf-IEs from culture showed that asexual stages appeared similar to Pf and Pv-IEs in the in vivo spleen sections (**Fig 1C**) and was used to validate the identification and staging of Pf-IEs between microscopists (**S4C Fig**). Pigmented phagocytes and phagocytosed parasites were observed on Giemsa-stained sections in both Pv and Pf and were clearly distinguishable from non-phagocytosed intact IEs by the presence of dispersed dark pigment or engulfed parasites throughout the cytoplasm of larger more intensely stained nucleated cells (**Fig 1D**). In PvAMA1-stained spleen sections (**Fig 2A, S4D Fig**), PvAMA1^+^ IEs often appeared non-phagocytosed and were distinguishable from parasite pigment. The non-phagocytosed PvAMA1^+^ IE-density in a typical spleen was similar to that of Pv mature asexual stages on matching Giemsa-stained sections (0.39% versus 0.29%, respectively, patient #1) [29], further confirming previous findings [29], and the accuracy of Giemsa-based quantifications and the specificity of PvAMA1-based staining. As reported in Pf [29], transmission electron micrographs taken from Pv-infected spleens also showed non-phagocytosed IEs (**Fig 2B**). CD68-based staining confirmed that a substantial proportion of Pv and Pf-IEs in the spleen were localised outside macrophages (**Fig 2C**), in line with previous results [29] and CD68 stains in ex vivo Pf-perfused spleens [28]. Taken together, histology, immunohistochemistry, and electron microscopy robustly showed the presence and ability to architecturally stage non-phagocytosed IEs in 15 of 17 spleens that could be analysed, opening the way to the comparison of parasite and reticulocyte densities/localizations in spleens and in peripheral blood.

#### Marked accumulation of asexual Pv and Pf in the spleen

**Fig 3:**
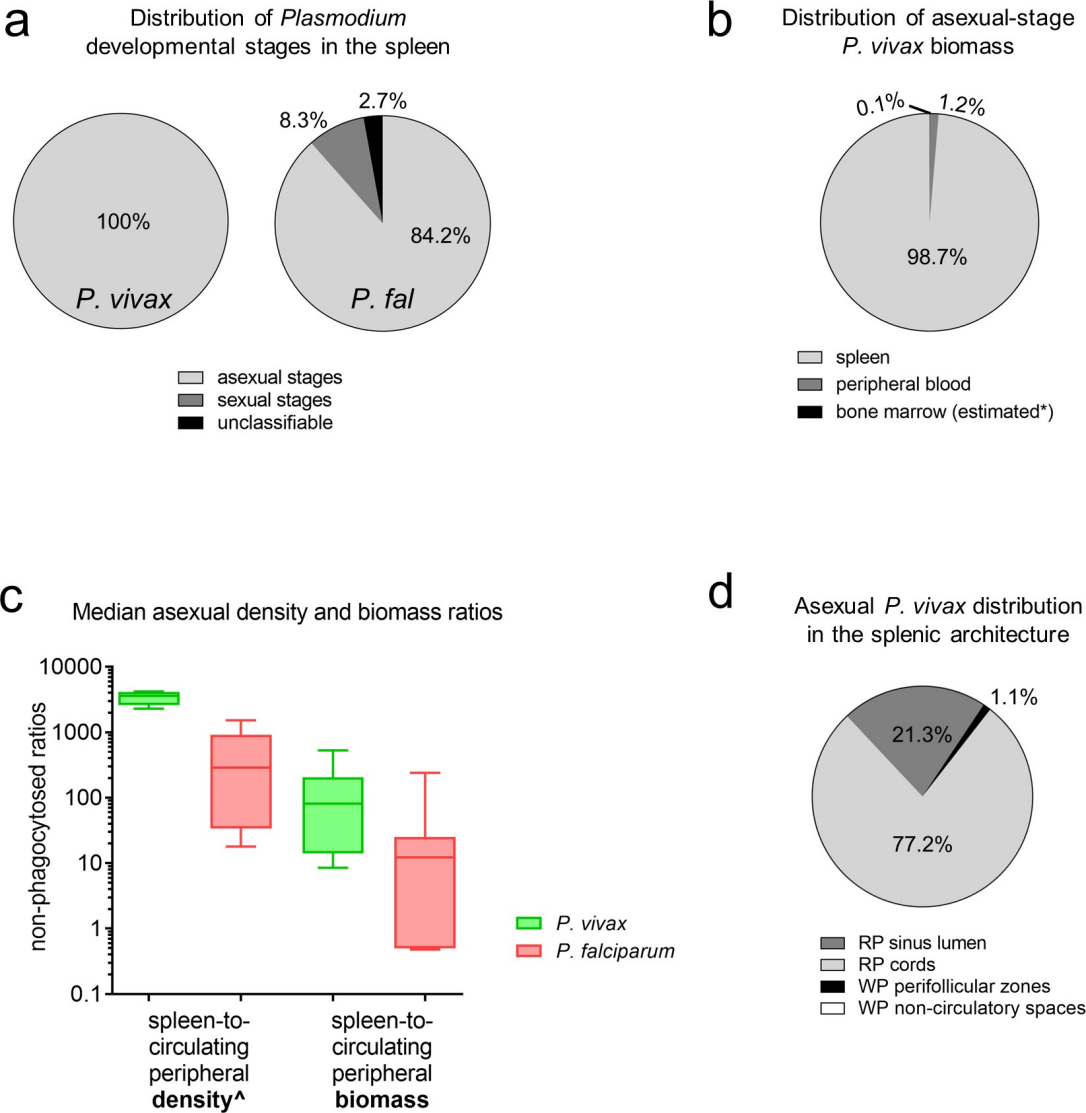
Splenic accumulation and distribution of non-phagocytosed IEs in cohort patients. Non-phagocytosed parasites in peripheral blood and spleen sections were counted by microscopy using an Olympus CX31 microscope. Stages were categorised into asexual, sexual, and unclassifiable for Pv (*n* = 6) and Pf (*n* = 9). In Pv and Pf, all individuals had asexual stages present. Sexual stages were reported in 1 Pv- and 6 Pf-infected individuals, and unclassifiable stages reported in 2 Pv- and 6 Pf-infected individuals, with the median distributions summarised as shown (a). As an additional comparator to the spleen, Pv biomass in the bone marrow was conservatively estimated based on a previous human study comparing relative Pv parasitaemia in the bone marrow to peripheral blood [20] (*). The median percentage of non-phagocytosed asexual Pv biomass found in the spleen, peripheral blood, and bone marrow was calculated (b). The median estimated non-phagocytosed spleen-to-peripheral asexual parasite density and biomass ratios were compared between 6 Pv and 7 Pf (c) (^reported previously [29]). The median distribution of asexual Pv stages in the splenic architecture was categorised into those found in the RP cords and sinus lumens, and WP non-circulatory spaces and perifollicular zones (d). Two individuals had <0.3% of asexual Pv in WP non-circulatory spaces. In panels a and d, the sum of segments in each pie chart may not add up to 100% due to medians in one of the groups being zero despite some individuals having values >0%. In panel c, medians, interquartile ranges, and ranges are shown, compared using the Mann–Whitney test (*p*-value <0.05 considered significant). IE, infected erythrocyte; Pf, *Plasmodium falciparum*; Pv, *Plasmodium vivax*; RP, red-pulp; WP, white-pulp.

Parasitaemia was determined under the optical microscope by counting IEs on Giemsa-stained smears from peripheral blood and tissue sections from spleens (see Methods for details). Results on the accumulation of asexual-stage Pv and Pf IEs in the spleen have been reported previously [29]. Briefly, the majority of non-phagocytosed IEs in infected spleens were asexual stages with a medians of 100% (IQR: 98.2% to 100%) in Pv and 84.2% (IQR: 58.7% to 90.9%) in Pf (**Fig 3A**). Sexual and unclassifiable stages were present to a greater extent in Pf than Pv (**Fig 3A**). We observed marked splenic tropism of asexual stages, whereby non-phagocytosed asexual parasitaemias were significantly higher in spleen tissue compared to circulating peripheral blood for both Pv (geometric mean 1.03% [95% CI: 0.480% to 2.19%] versus 0.0003% [95% CI: 0.0001% to 0.0006%], *p* = 0.03) and Pf (1.53% [95% CI: 0.678% to 3.43%] versus 0.007% [95% CI: 0.001% to 0.044%], *p* = 0.02) [29]. All Pv infections had PvAMA1+ IEs present (**S4D Fig**) suggesting the presence of mature asexual Pv stages. In Giemsa-stained spleen sections from 2 individuals with Pv infection, 14.7% and 16.8% of asexual stages in the spleen were identified as schizonts, while the remainder were asexual rings and trophozoites. These proportions are consistent with the upper-end duration in which Pv rings/trophozoites (42 to 45.6 hours) and schizonts (2.4 to 6 hours) exist in ex vivo Pv studies [64,65] when expressed as a percentage of the 48-hour lifecycle (88% to 95% rings/trophozoites and 5% to 12% schizonts, respectively). In Pf, the median proportion of rings/trophozoites and schizonts in the spleen were 90% (IQR: 67.3 to 100) and 10% (IQR: 0 to 32.7), respectively [29]. All asexual stages found in peripheral circulation were rings/trophozoites in Pv and rings only in Pf.

#### The bulk of asexual Pv biomass is in the spleen

Splenic and peripheral parasite biomasses were estimated based on spleen weight and total blood volume, respectively (see Methods for details). We have previously reported [29] that in Pv-infected individuals, a median of 2.74 × 10^9^ (95% CI: 0.26 × 10^9^ to 25.6 × 10^9^) non-phagocytosed asexual Pv IEs were estimated to be in the spleen, significantly higher than the 0.04 × 10^9^ Pv-IEs (95% CI: 0.03 × 10^9^ to 0.3 × 10^9^) estimated to be circulating in peripheral blood (*p* = 0.03). Asexual biomass estimates in Pf were not significantly different in the spleen (median 8.56 × 10^9^ IEs [95% CI: 2.18 × 10^9^ to 28.9 × 10^9^]) compared to circulating peripheral blood (0.35 × 10^9^ IEs [95% CI:0 to 3.99 × 10^9^], *p* = 0.10) [29]. Our conservative estimates of asexual Pv biomass in the bone marrow suggest that this tissue does not appear to be a major contributor to total-body Pv biomass (median of 0.1% [IQR: 0.04% to 0.21%]; **Fig 3B**), at least for asexual-stage Pv. The spleen is the predominant compartment for asexual Pv biomass (98.7% [IQR: 95.1% to 98.9%]), followed by a small intravascular component (1.2% [IQR: 1% to 5.8%]; **Fig 3B**) where Pv transmission stages are accessible to the vector.

#### Greater splenic tropism of asexual Pv than Pf

We have previously reported [29] that splenic asexual Pv-IE density was 3,590 times (IQR: 2,600 to 4,130) higher than circulating in peripheral blood, significantly greater than the estimated spleen-to-circulating blood density ratio in Pf infections (median 289 [IQR: 33.9 to 918]; **Fig 3C**). Total biomass ratios in these compartments indicated that splenic asexual Pv biomass was a median 81 times (IQR: 14 to 205) higher than circulating in peripheral blood, while the median spleen-to-circulating peripheral blood biomass ratio in Pf was 12 (IQR: 0.5 to 25; **Fig 3C**). Although such ratios do not include Pf-IEs sequestered within non-splenic blood vessels, approximately 10-fold higher ratios observed in Pv suggest greater splenic tropism with infection by this species.

#### Culturable splenic asexual IEs are viable and are mostly in the red-pulp cords

To assess the viability of IEs in human spleens, the ability of splenic *Plasmodium* to grow ex vivo was examined by relative changes in asexual stage frequency in concordance with the known parasite lifecycles. Growth of splenic parasites was observed in 8 of 12 Pf-infected individuals (**S1 Table**), in line with previous findings [29], including an absence of growth from peripheral blood (patient #9). No Pv growth/maturation was observed in spleen blood from the 5 Pv-infected individuals with <0.1% parasitaemia, cultured without reticulocyte enrichment.

The localization of non-phagocytosed asexual IEs was categorised into 4 splenic zones. A median of 77.2% (IQR: 15.9% to 38.8%) and 21.3% (IQR: 60.8% to 81.6%) of asexual Pv-IEs were found in the red-pulp cords and sinus lumens, respectively (**Fig 3D**).

Taken together, asymptomatic Pv infection is characterised by intense log-scale accumulation of intact asexual parasites in the spleen [29]. Splenic tropism was especially predominant in Pv, with young and mature asexual stages proportional to their lifecycle in vivo highly suggestive of an endosplenic Pv replication cycle.

### Could the magnitude of Pv-IE accumulation in the spleen arise solely from retention of peripherally produced parasites?

Our findings indicate an intrasplenic Pv reservoir 80-fold greater than the whole parasite population in circulation, and Pv parasite densities thousands of times higher in the spleen compared to peripheral blood. We extend our previous calculations [29] to determine if the magnitude of splenic tropism could be achieved from the retention of peripherally produced Pv-IEs alone. Assuming an average adult blood volume of 4 litres, a splenic blood volume of 15 mL/100 g of spleen tissue (determined from ex vivo flushing experiments), and an initial input of 0.0003% peripheral Pv parasitaemia (as observed in our cohort), a 280 g spleen (the median weight of Pv-infected spleens in our cohort) containing 42 mL of blood (≈21 mL of RBCs) with 90% splenic retention of IEs would have a splenic parasitaemia of 0.026% before the next lifecycle (86 times higher than the initial input in circulation). This is nearly 40 times lower than the observed intrasplenic Pv parasitaemia of approximately 1% in our cohort. To accommodate ongoing replication within peripheral blood, we added a multiplication rate to the calculation. The prevailing effective multiplication rate in chronic asymptomatic malaria is considered to be 1 per 48-hour lifecycle after taking into account invasion efficiency and parasite clearance. Even assuming a multiplication rate of 10 in peripheral blood (which is >50% efficiency for Pv in 1 cycle) [66], 99% splenic retention of IEs, and zero destruction by splenic macrophages or other intrasplenic processes, the resulting intrasplenic Pv parasitaemia of 0.28% is still far from the approximately 1% observed in our cohort. Taken together, the density and biomass of Pv in the spleen cannot be explained numerically by parasite replication events in the circulation alone. One potential explanation for this discrepancy is the presence of an endosplenic asexual Pv lifecycle, which we hypothesised would be supported by a splenic reservoir of immature reticulocytes.

### The human spleen is a pool for immature CD71^+^ reticulocytes that are targeted by Pv for invasion

**Fig 4:**
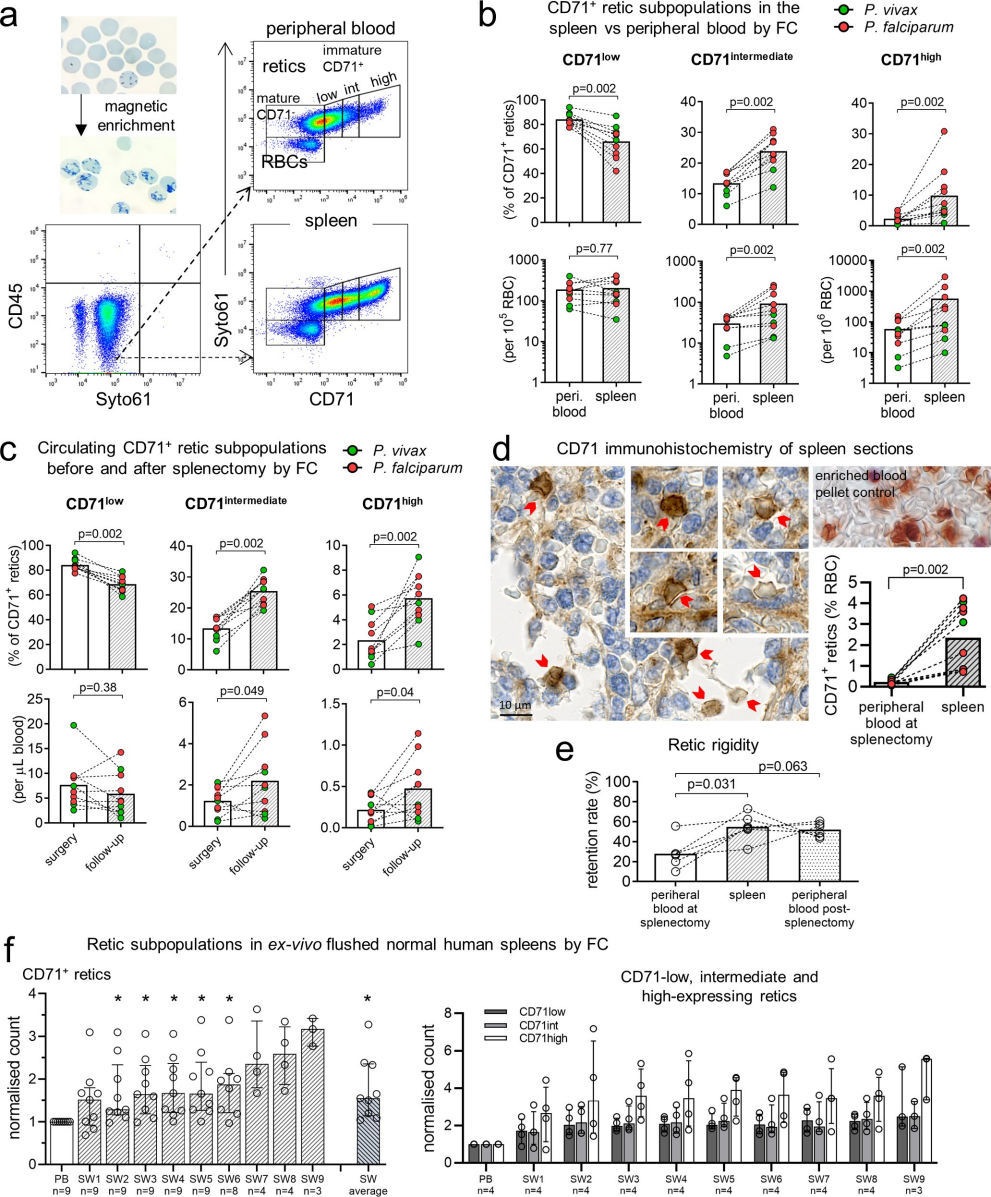
Increased density of immature CD71^+^ reticulocytes in human spleens. Samples were magnetically enriched for reticulocytes (retics) and phenotyped by CD71 FC (a). The median retic purity in enriched samples was 95.8% [IQR: 94.3%–98.5%] for PB and 56.8% [IQR: 53.4%–72.6%] for sliced-spleen blood. Numbers as a percentage of CD71^+^ retics and per 10^5^ or 10^6^ RBCs were determined for CD71 low-, intermediate- and high-expressing retics in PB and the spleen (b) (*n* = 10 pairs). In addition, CD71^+^ retic populations in PB several months after splenectomy (median post-splenectomy retic enrichment purity of 84.0% [IQR: 71.9%–86.6%]) was compared to PB at surgery (*n* = 10 pairs), presented as a percentage of CD71^+^ retics and as absolute counts per μL blood (calculated based on automated RBC counts that were available for PB) (c). CD71^+^ retics were visualised on spleen section by CD71 immunohistochemistry (patient #4 section shown) and counted based on guidelines derived from CD71 staining of a retic-enriched blood pellet (d). CD71^+^ retics concentrations in the spleen by immunohistochemistry was compared to CD71^+^ retic concentrations in PB determined by FC (*n* = 10 pairs) (d). Peripheral and splenic retic deformability in 6 patients was determined by microsphiltration and presented as retention rates (e). In France, ex vivo flushing of 9 uninfected control spleens were performed (f). For each of the first 5 spleens, a PB fraction was collected either from the basilic or splenic vein, followed by 5–6 consecutive SW fractions from 350–500 mL of flushing buffer. With initial results indicating a trend towards higher proportions of immature retics in the last SW fractions, the volume of flushing buffer was increased to 500–650 mL for each of the next 4 spleens, resulting in 8–9 SW fractions being collected. Immature CD71^+^ retics were quantitated in each fraction by FC (see S2D Fig), normalised, and compared to PB (f, left). In the last 4 spleens, retics were phenotyped into CD71 negative, low-, intermediate-, and high-expression (f, right). Paired datapoints are connected by lines. Bars in all panels represent medians. Error bars in f are interquartile ranges. The Wilcoxon test was used for all statistical comparisons (*p < 0.05 considered statistically significant). FC, flow cytometry; PB, peripheral blood; RBC, red blood cell; SW, spleen wash.

Reticulocytes in enriched peripheral and sliced-spleen blood were categorised into CD71 subpopulations by flow cytometry (**Fig 4A**) in a subset of the patients splenectomised due to trauma, all with asymptomatic *Plasmodium* infection (**S2 Table**). The percentage and number of immature CD71 intermediate- and high-expressing reticulocytes were significantly higher in sliced-spleen blood relative to matching peripheral blood (all *p* = 0.002; **Fig 4B**). In the 2 to 5 months following splenectomy, both the percentages (*p* = 0.002) and absolute numbers (*p* < 0.05) of immature CD71 intermediate- and high-expressing reticulocytes in peripheral blood were significantly higher compared to values measured on the day of splenectomy (**Fig 4C**).

To support flow cytometry findings, spleen sections from the same patients (**S2 Table**) were stained with CD71 by IHC to directly visualise and count immature CD71^+^ reticulocytes (**Fig 4D, S2C Fig**). Here, infected spleens had a median immature CD71^+^ reticulocyte concentration of 2.4% of RBCs [IQR: 0.8% to 3.9%], 11 times higher than the median in matching peripheral blood by flow cytometry (0.22% [IQR: 0.12% to 0.33%], *p* = 0.002; **Fig 4D**). To provide insight into potential mechanisms of splenic accumulation, a microsphiltration assay was used to examine reticulocyte deformability. Reticulocytes in the spleen and post-splenectomy peripheral blood were found to be more rigid compared to those in peripheral blood on the day of splenectomy (*p* = 0.03 and *p* = 0.06, respectively; **Fig 4E)**, suggesting biomechanical retention upstream of interendothelial slits in the red-pulp may be a contributing mechanism of splenic reticulocyte accumulation.

To confirm that the splenic reticulocyte reservoir was not only restricted to infected spleens, reticulocytes were examined in uninfected control spleens by ex vivo flushing from 9 patients undergoing elective spleno-pancreatectomy in France (**S3 Table**). Here, normalised immature CD71^+^ reticulocyte counts determined by flow cytometry (**S2D Fig**) were significantly higher in fractions representing splenic content in comparison to peripheral blood (**Fig 4F**, left). In 4 of the spleens where examination of CD71 subpopulations was performed, an enrichment of CD71-negative, low-, intermediate-, and high-expressing reticulocytes was clearly apparent in splenic effluents compared to peripheral blood (**Fig 4F**, right).

In summary, immature CD71^+^ reticulocytes, the target cells for Pv invasion, accumulate in the human spleen physiologically and during infection. Following splenectomy, this cell population is present at higher concentrations in peripheral blood. These findings suggest that reticulocytes naturally accumulate in the human spleen.

### Colocalization of non-phagocytosed IEs and immature CD71^+^ reticulocytes in specific splenic compartments suggest an endosplenic Pv lifecycle

**Fig 5:**
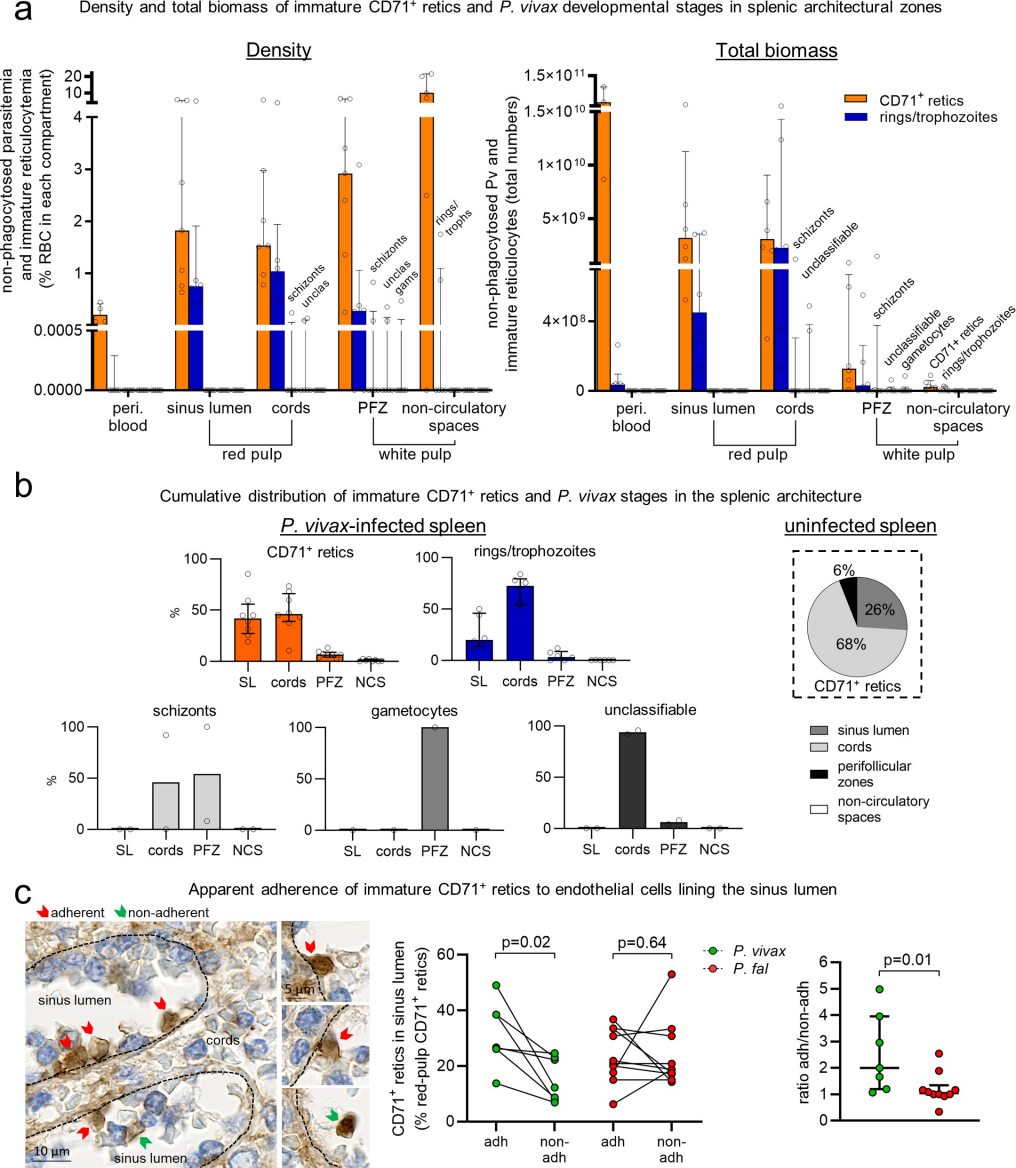
Immature CD71^+^ reticulocytes (retics) and non-phagocytosed Pv-IEs colocalise in specific splenic compartments. The density and total biomass of CD71^+^ retics and non-phagocytosed Pv developmental stages (*n =* 6) were determined in the red-pulp SL, cords, PFZ, and white-pulp NCS (a). Non-phagocytosed Pv parasites were categorised into 3 groups (rings/trophozoites, schizonts, gametocytes), and a fourth group of those with unclassifiable stages. The cumulative distribution of CD71^+^ retics and *Plasmodium* developmental stages in the splenic architecture was calculated in individuals in whom intrasplenic parasites were visualised (b, left). The distribution of CD71^+^ retics in an uninfected spleen is also shown (b, right). In the splenic SL, a large proportion of CD71^+^ retics were apparently adherent to endothelial cells on the luminal side as observed by CD71 immunohistochemistry (c, left, representative image from Pv patient #4). The number of those apparently adherent (adh) and non-adherent (non-adh) were expressed as a percentage of red-pulp CD71^+^ retics in Pv as well as Pf, and compared as paired data (connected lines) using the Wilcoxon test (c, middle). The ratio of adherent-to-non-adherent CD71+ retics in the SL was compared between Pv and Pf using the Mann–Whitney test (c, right). A *p*-value <0.05 was considered significant. Data in a, b (left), and c (right) are individual datapoints with median and interquartile range. adh, adherent; NCS, non-circulatory space; non-adh, non-adherent; Pf, *Plasmodium falciparum*; PFZ, perifollicular zone; Pv, *Plasmodium vivax*; RBC, red blood cell; SL, sinus lumen.

The locations of non-phagocytosed IE stages and immature CD71^+^ reticulocytes in the splenic microcirculatory compartments were characterised by histopathology. All asexual Pv developmental stages were observed in the spleen (**Fig 5**). Immature CD71^+^ reticulocytes accumulated in all splenic compartments compared to peripheral blood and were expectedly more concentrated in each of the compartments than any of the *Plasmodium* stages (**Fig 5A**, left). These cells predominated in the red-pulp cords and sinus lumens in Pv-infected spleens (median of 46% and 42%, respectively; **Fig 5B**).

In Pv infection, rings/trophozoites predominated over other Pv stages in each of the splenic compartments (**Fig 5A**). Overall, a median of 73% of Pv rings/trophozoites were observed in the cords and 20% were in the sinus lumens (**Fig 5B**). As such nearly all splenic Pv rings/trophozoites (93%) colocalised in the red-pulp with immature CD71^+^ reticulocytes (88% of which were in the red pulp cords and sinus lumens). Pv schizonts in the spleen were found at lower densities and were absent in peripheral blood (**Fig 5A**). Their distribution varied widely with medians of 54% in the red-pulp cords and 46% in the PFZ (**Fig 5B**). The PFZ was the only compartment found to contain the very few Pv gametocytes that were observed in the spleen of a single individual (**Fig 5A and 5B**).

The opportunity to histologically evaluate an uninfected spleen in our Papuan cohort revealed an apparent enrichment of immature CD71^+^ reticulocytes in the splenic sinus lumens during Pv (26% in control versus 42% in Pv; **Fig 5B**). Closer examination of infected CD71-stained spleen sections uncovered a considerable proportion of immature CD71^+^ reticulocytes that were in close contact with and apparently adherent to endothelial cells lining the luminal side of the sinuses (**Fig 5C**, left). Counting of apparently adherent versus non-adherent reticulocytes in these spaces indicated that a significantly higher proportion of immature CD71^+^ reticulocytes in the red-pulp were apparently adherent in the sinus lumens of Pv-infected spleens (*p* = 0.02), while proportions of adherent and non-adherent sinus lumen reticulocytes in Pf were generally similar (*p* = 0.64; **Fig 5C**, middle). Ratios of adherent-to-non-adherent immature CD71^+^ reticulocytes showed twice as many apparently adherent cells in Pv (IQR: 1.2 to 4.0), significantly higher than the 1:1 ratio in Pf (IQR: 1 to 1.3, *p* = 0.01; **Fig 5C**, right).

Taken together, the accumulation and colocalization of all asexual-stage malaria parasites and immature CD71^+^ reticulocytes in the splenic red-pulp in asymptomatic Pv infections is strongly suggestive of an endosplenic asexual lifecycle, of greater magnitude in Pv. The greater proportion of immature CD71^+^ reticulocytes apparently adhering in the sinus lumen in Pv may reflect an additional population of target cells for invasion, statically located in the direct path of merozoites released from rupturing Pv schizonts upstream in the cords and PFZ. Differences in the splenic compartmentalization and numbers of schizonts and gametocytes between Pv and Pf may reflect different pathophysiological mechanisms contributing to their splenic retention and modes of pathogenesis/transmission.

## Discussion

The swollen spleen of nonfebrile humans exposed to *Plasmodium* contains a substantial hidden biomass of intact IEs, with densities hundreds to thousands of times higher than in circulating peripheral blood [29]. Expanding these analyses with additional experiments and patients has uncovered strong evidence to support the existence of a cryptic endosplenic lifecycle in asymptomatic Pv infections. Both immature CD71^+^ reticulocytes and asexual-stage Pv were concentrated and colocalised predominantly in the splenic red-pulp, consistent with a role for the human spleen in both reticulocyte maturation and Pv growth and replication. The distinct distribution of schizonts and greater adherence of sinus lumen reticulocytes in Pv-infected spleens, are consistent with recently described spleen-specific Pv cytoadherence [67,68], and suggest Pv-specific adaptations to survive in this organ. Vivax malaria should be considered predominantly an infection of the reticulocyte-rich spleen, with secondary involvement of the intravascular compartment. While important for transmission and pathogenesis, the circulating compartment is not the principal site for parasite biomass and parasite interactions with cells mediating immune responses.

Our findings indicate an intrasplenic Pv pool 80-fold greater than the whole parasite population in circulation. This substantial difference cannot be explained numerically by parasite replication events in the circulation alone, with our deductive calculations (outlined in the Results) indicating that splenic retention of 90% of Pv-IEs from circulation would achieve an intrasplenic parasitaemia of 0.03%, 40 times lower than what we observed in our cohort. Even adding a parasite multiplication rate in peripheral blood of 10 to the calculation, the resulting intrasplenic Pv parasitaemia is still 4-fold lower than observed in vivo. Of note, this assumed multiplication rate of 10 is likely an overestimate in light of our observation that a proportion of retained Pv-IEs are phagocytosed. A cryptic asexual Pv lifecycle outside the peripheral blood is therefore by far the most robust explanation of our observations.

Previous studies in animals [33–37] and humans [38] have suggested accumulation of total reticulocytes in the spleen. Here, we show that the human spleen is a hitherto unrecognised reservoir for highly immature CD71 intermediate- and high-expressing reticulocytes. Their increase in peripheral blood for months after splenectomy provides further indication of their retention in the human spleen. The greater rigidity of immature splenic reticulocytes was evident by the reduced deformability of splenic compared to circulating reticulocytes, and along with their predominance in the splenic cords is consistent with a biomechanical component to their retention in the spleen. Immature reticulocytes also have adhesive capacity in vitro [30], and their apparent adherence to sinus lumen endothelial cells in our cohort suggests cytoadhesion as an additional mechanism by which immature CD71^+^ reticulocytes accumulate in the spleen. Further non-histological studies are warranted to confirm the apparent adherence of reticulocytes in the sinus lumen. Our observations in uninfected controls also suggest that the human spleen is a natural physiological niche for maturation of immature reticulocytes. Human erythropoiesis occurs physiologically in the bone marrow but in contrast to murine models [69,70], is extramedullary only in rare disorders [71,72]. Whether erythropoiesis occurs in the malarial spleen remains unclear and was not specifically examined in this study.

Apart from the increased rigidity of late/segmenting Pv schizonts [73] (and probably very early rings up to 3 hours post-invasion [31]), the majority of circulating Pv parasites are generally deformable [74,75] suggesting these stages are less likely to be retained in the spleen biomechanically. The formation of Pv rosettes reduces their deformability and may provide a physical shield against the cell-mediated immune response [73] while enhancing splenic accumulation. However, structures resembling Pv rosettes have not been observed in tissues in vivo and were not obvious in the fixed spleen samples in this study. Pv-infected reticulocytes are known to cytoadhere in vitro to human splenic fibroblasts that are found in the red-pulp cords [67,68,76], and such cytoadherence may explain the splenic retention of a large proportion of Pv in our cohort. We speculate that a similar mechanism may be underlying the accumulation of Pv schizonts in the PFZ of the fast circulation. The expression of VIR proteins on the surface of Pv-IEs have been shown to facilitate binding to intercellular adhesion molecule-1 [76,77] and may be linked to Pv cytoadherence in the spleen, yet the exact interactions remain unclear and warrant further investigation.

Our findings on the splenic accumulation and colocalization of different asexual stages of Pv and immature CD71^+^ reticulocytes raise fascinating hypotheses regarding the biology of an endosplenic asexual Pv lifecycle. Firstly, the small proportion of schizonts (15% to 17%) relative to rings/trophozoites (85% to 100%) in Pv-infected spleens is in line with their short duration in the Pv lifecycle (2.4 to 6 hours), with schizonts estimated to represent 5% to 12% of the 48-hour Pv lifecycle [64,65]. Secondly, compared to the vasculature, reduced blood flow and shear stress in the slow open circulation of the splenic cords plausibly offer a more suitable environment for successful reticulocyte invasion, the exact compartment where we observed 46% of Pv schizonts and 73% of earlier stages colocalised with close to half of the immature reticulocytes in the spleen. Segmenting schizonts biomechanically trapped at the cordal-side of inter-endothelial slits will rupture and provide the opportunity for merozoites to rapidly invade surrounding immature reticulocytes that are also trapped in the vicinity. In addition, we speculate that the small merozoites may readily cross inter-endothelial slits and invade the second line of reticulocytes cytoadhering in the sinus lumens just downstream from these slits. Thus, the splenic red-pulp shapes a niche for *Plasmodium* development, with its dense population of immature reticulocytes especially favourable for Pv [16,31]. That schizont-stage Pv become more fragile after successfully traversing tight slits [74] is speculatively a favourable adaptation that may accelerate rupture and increase the chance of invasion of adjacent cytoadhering static immature reticulocytes in the sinus lumens.

Thirdly, we speculate that the schizonts located in the fast-circulation PFZ may serve as a second source of merozoites to invade both adherent and non-adherent immature reticulocytes that accumulate downstream in the sinus lumens. A lower chance of closely interacting with immune cells is an advantage of being in these compartments compared to the cords, but with a faster flow rate similar to peripheral circulation, it is possible that invasion events are less likely to be successful in these spaces despite an equal number of immature reticulocytes being available as the cords. This would be supported by the disproportionally large number of early asexual stages, representing successfully invaded reticulocytes, in the cords (73%) compared to sinus lumens (20%). Reticulocytes infected in the sinus lumens will eventually exit the spleen and circulate. We therefore hypothesise that maintaining a presence in the fast circulation of the spleen may partly be an adaption to maintain transmission, whereby a proportion of invaded reticulocytes are destined to circulate as gametocytes. Presence in compartments of the fast circulation may also allow Pv to survive during periods of immune activation or destabilised tolerance in the splenic cords. In settings such as acute infection, where the immunotolerant conditions reflected in our cohort is not yet acquired or has been lost, a greater proportion of Pv circulate. While these hypotheses remain speculative, our findings to date are consistent with Pv having evolved multiple mechanisms to maximise their survival and replication in the spleen.

Reports in humans [19–22] and findings in a splenectomised nonhuman primate model [50] have shown that the bone marrow, also rich in the immature CD71^+^ reticulocytes [31], and known to be a niche for Pf sexual stages [23,78,79], is recognised as an additional compartment for asexual and sexual Pv accumulation. It was not possible to examine bone marrow in our splenectomy cohort. Our conservative estimates based on a 1:1 ratio of bone marrow-to-circulating parasitaemia in uncomplicated acute Pv [20] suggest that the bone marrow does not appear to be a major contributor to total-body Pv biomass in asymptomatic infections, at least for asexual stages. Findings in the splenectomised primate model implicate the bone marrow as an important space for Pv gametocyte development [50] which may explain the rarity of these stages in spleens from our cohort. However, the degree to which asexual and sexual Pv may additionally infect the bone marrow in asymptomatic infections remains unknown.

With the entire intraerythrocytic asexual Pv lifecycle likely taking place predominantly in the spleen, our study provides a new framework to explore innate and adaptive immune responses to Pv and Pf. Human studies on the role of the spleen in malaria immunity are urgently needed to inform vaccine development. We hypothesise that immunotolerance to *Plasmodium* is developed in the cords of the spleen, induced by sustained high antigen loads. This is evidenced by a large number of pigmented phagocytes and non-phagocytosed IEs in our cohort. We speculate that, in addition to phagocytosis of active parasites, a proportion of the pigment engulfed by phagocytes may come from rupturing schizonts [80] and may also represent remnants from past infections [81]. Nevertheless, the predominance of pigmented phagocytes in all spleens and visualisation of phagocytosed parasites in both Pv and Pf infections provide direct in vivo evidence of a major concurrent host-protective role for the spleen in asymptomatic human *Plasmodium* infection and direct in vivo evidence for the long-hypothesised clearance of untreated parasites. These data complement previous observations on the central role of the spleen in clearing parasites following treatment with antimalarials [26], postmortem data from fatal Pf malaria series [23,25,27], the majority of which had received antimalarial treatment prior to death, and the biomechanical retention and/or clearance of treated [28] and untreated [44,59] IEs seen in Pf ex vivo spleen studies.

Our study has several limitations. We report parasitaemia findings in semi-immune individuals with chronic asymptomatic infection, the majority with enlarged spleens. Our study also had a small sample size. These limit the interpretation and generalizability of our findings and warrant larger studies including in acute symptomatic malaria. Splenic trauma or rupture are rare during clinical malaria and performing splenic studies in untreated clinical disease may require indirect, noninvasive evaluation. Recent positron emission tomography and magnetic resonance imaging in experimental human volunteer infection studies indicate greater splenic tropism and metabolic activity in early Pv infection compared to Pf [82], suggesting that splenic accumulation of Pv occurs even early in infection. Our cohort requiring splenectomy may also not be representative of the wider population. The inability to maintain Pv in continuous culture and its fastidious growth requirements limited our ability to detect Pv growth and confirm viability of the intact non-phagocytosed splenic Pv-IEs. However, ex vivo parasite maturation confirmed the viability of non-phagocytosed intrasplenic Pf, including instances of growth from splenic but not peripheral blood. Even in the most optimal circumstances (immediate processing directly from venepuncture), a significant proportion of Pv perish under ex vivo conditions, thus requiring a higher initial parasitaemia (>0.1%) than found in our spleen blood eluates to ensure enough parasites survive to enable accurate experimental observations [83].

In conclusion, our findings provide a major contribution to the understanding of malaria biology and pathology. The spleen sustains a very large biomass of non-phagocytosed asexual Pv parasites, concurrent with the increased availability of immature CD71^+^ reticulocytes, both colocalised in splenic environments where invasion-reinvasion events would form a cryptic endosplenic lifecycle. Like Pf, quantifying circulating parasitaemia alone will significantly underestimate total-body Pv biomass in asymptomatic infections. This hidden biomass is likely a significant contributor to anaemia and challenges the assumptions underlying prevailing mathematical models of malarial anaemia derived from circulating parasitaemia and the proportions of uninfected-to-infected RBCs lost in chronic infections from both *Plasmodium* species [84,85], particularly so for Pv. Cross-talk between different parasite species/stages and immune cells are likely different in specific intrasplenic microenvironments compared to the circulation, with architectural distributions suggesting Pv may have evolved multiple mechanisms to maximise their survival and replication in the spleen.

## Supporting information

S1 Fig Spleen dissection and microscopist validations. Each spleen was weighed and recorded to nearest gram (a), then sliced in half using sterile disposal blades (b). One half of the spleen was sliced longitudinally (c) and used for downstream experiments, while the second half was processed into paraffinised spleen tissue blocks. A subset of *n =* 6 Giemsa-stained spleens were read by 2 expert microscopists to validate non-phagocytosed IE counts, including the ability to distinguish into the different splenic architectural zones and parasite stages (d). Each reader examined spleen sections from different spleen biopsies in the same patient. IE, infected erythrocyte; RBC, red blood cell.
(TIF)

S2 Fig Reticulocytes in the spleen and in peripheral blood. Heated and normal RBCs were used for quality control of microsphere tips and gated as shown (a). Retention rates (mean ± standard error) of >95% and <5% for heated and normal RBCs, respectively, satisfied minimum requirements (b). Immature CD71^+^ reticulocytes (retics) in *P*. *falciparum*- and *P*. *vivax*-infected spleens were stained by immunohistochemistry with antibodies against the CD71 transferrin receptor; additional examples of retics are shown (c). A 3-colour flow cytometry stain was used to quantitate retics in SW obtained from the flushing of normal spleens in France, identified as CD45^−^thiazole-orange^+^ cells, with or without CD71 expression (d). A 3-colour flow cytometry panel was used to phenotype retics in the Indonesian cohort, identified as CD45^−^SYTO61^+^ cells with or without CD71 (e). Numbers per thousand RBCs were determined for total, immature CD71^+^ and mature CD71^−^ retics in PB and sliced SB samples (*n* = 10 pairs, Wilcoxon test, f). Bars represent medians. FS, forward scatter; PB, peripheral blood; RBC, red blood cell; SB, spleen blood; SW, spleen wash; TO, thiazole-orange.
(TIF)

S3 Fig Images of spleens. Each patient’s spleen was photographed for macroscopic records. Images not available for patient #13. Spleen weights presented in grams.
(PDF)

S4 Fig Malaria parasites in spleen sections and microscopy validations. Representative images of Giemsa-stained spleen sections from *P*. *vivax*- (a, patient #1) and *P*. *falciparum*-infected spleens (b, patient #20) showing non-phagocytosed IEs (red arrowhead), pigmented phagocytes (green arrowheads) in the cords and sinus lumen. Giemsa-stained sections from the ex vivo perfused spleen (Fig 1C) were read by 2 expert microscopists (c). Reading included 5 fields of RP comprising cords and sinus lumen (sl), and 5 fields of WP, with the resulting counts in close agreement (<15% variation from the mean of readings). Additional images from immunohistochemical stains using antibodies against PvAMA1 were taken from several *P*. *vivax*-infected spleen sections showing merozoites/mature stages staining red (d). IE, infected erythrocyte; PvAMA1, Pv apical membrane antigen-1; RP, red-pulp; sl, sinus lumen; WP, white-pulp.
(TIF)

S1 Table Ex vivo growth of peripheral and splenic *Plasmodium* parasites. (DOCX)

S2 Table Baseline characteristics of patients with reticulocytes evaluated by flow cytometry. (DOCX)

S3 Table Baseline characteristics of *n =* 9 spleno-pancreatectomy patients in France. (DOCX)

S4 Table Full blood counts at surgery. (DOCX)

## Abbreviations

ACD: acid citrate dextrose
EDTA: ethylenediaminetetraacetic acid
FFPE: formalin-fixed paraffin-embedded
gDNA: genomic DNA
HMS: Hyperreactive Malarial Splenomegaly
HPF: high-power field
HRP2: histidine-rich protein-2
IE: infected erythrocyte
IgM: immunoglobulin M
IHC: immunohistochemistry
NBF: neutral-buffered formalin
PBS: phosphate-buffered saline
PCR: polymerase chain reaction
Pf: *Plasmodium falciparum*
PFZ: perifollicular zone
pLDH: *Plasmodium* lactate dehydrogenase
Pv: *Plasmodium vivax*
PvAMA1: Pv apical membrane antigen-1
RBC: red blood cell
RSUD: Rumah-Sakit-Umum-Daerah
RT: room temperature
WBC: white blood cell
