# Correction: Evaluation of splenic accumulation and colocalization of immature reticulocytes and *Plasmodium vivax* in asymptomatic malaria: A prospective human splenectomy study

**DOI:** 10.1371/journal.pmed.1005254

**Published:** 2026-09-22

**Authors:** Steven Kho, Labibah Qotrunnada, Leo Leonardo, Benediktus Andries, Putu A. I. Wardani, Aurelie Fricot, Benoit Henry, David Hardy, Nur I. Margyaningsih, Dwi Apriyanti, Agatha M. Puspitasari, Pak Prayoga, Leily Trianty, Enny Kenangalem, Fabrice Chretien, Valentine Brousse, Innocent Safeukui, Hernando A. del Portillo, Carmen Fernandez-Becerra, Elamaran Meibalan, Matthias Marti, Ric N. Price, Tonia Woodberry, Papa A. Ndour, Bruce M. Russell, Tsin W. Yeo, Gabriela Minigo, Rintis Noviyanti, Jeanne R. Poespoprodjo, Nurjati C. Siregar, Pierre A. Buffet, Nicholas M. Anstey

This *PLOS Medicine* article [1] reports findings from an expanded study of 22 patients; data from 15 patients in this cohort were previously reported in [2] (cited as reference [29] in [1]). The upper left-hand panel of Fig 1a, when flipped horizontally and rotated 90˚, is a partially overlapping image of the same representative tissue section shown in the left-hand panel in Fig 1B of [2].

As such, the upper left-hand panel of Fig 1a reports material from [2], published in 2021 by Massachusetts Medical Society, which is not offered under a CC BY license and are therefore excluded from this article’s [1] license.

This clarification does not affect the results, conclusions, and overall scientific understanding.

The Data Availability statement in [1] is incorrect. The correct Data Availability statement is: All relevant data are within the manuscript, its Supporting Information files and in the following online repository: https://doi.org/10.6084/m9.figshare.32332452.
