# Supplementary figures and images for: Evaluation of splenic accumulation and colocalization of immature reticulocytes and Plasmodium vivax in asymptomatic malaria: A prospective human splenectomy study
Source: PLoS Med. 2021 May 26;18(5):e1003632. doi: 10.1371/journal.pmed.1003632 (PMC8154101; doi:10.1371/journal.pmed.1003632)

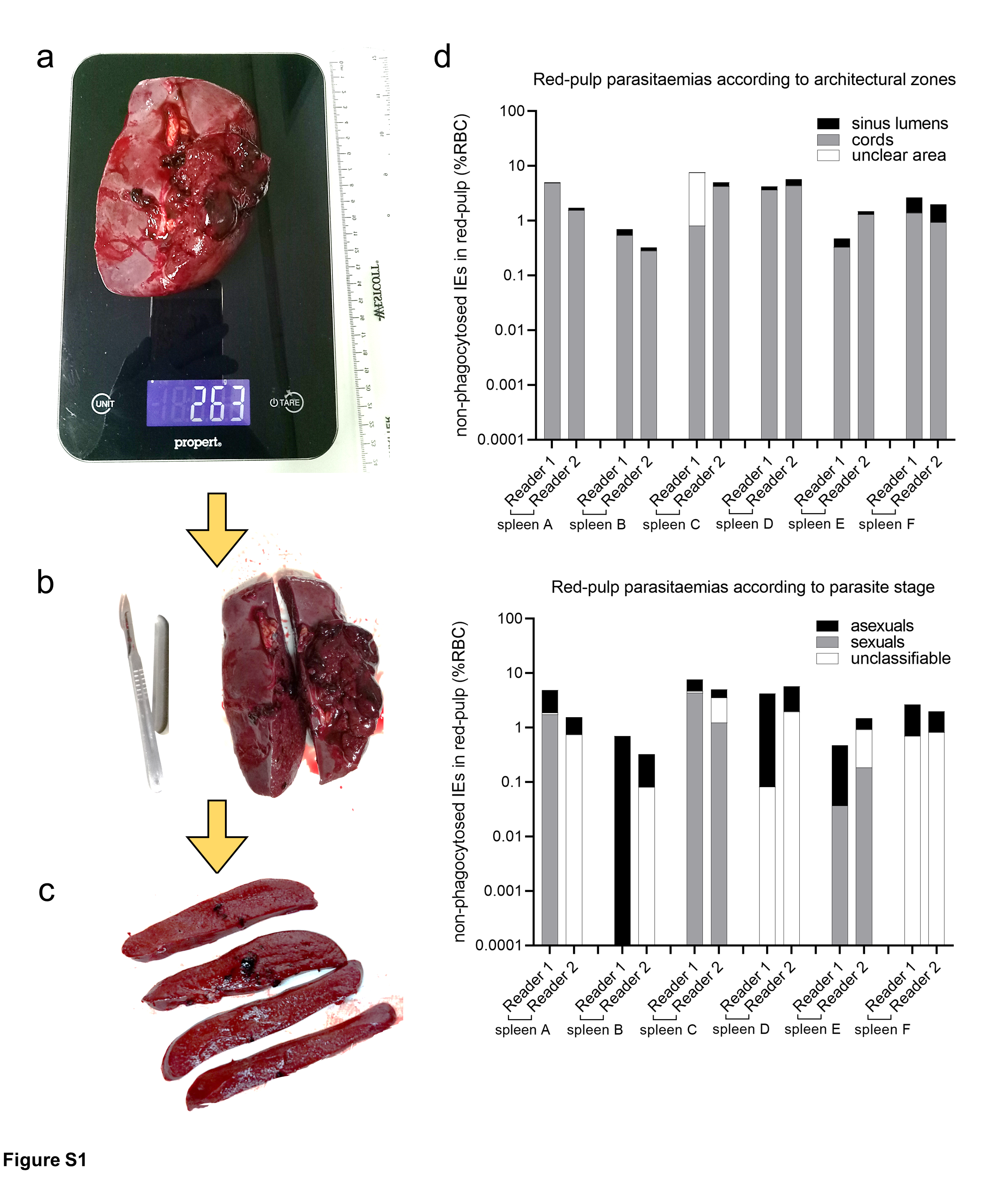

Supplement: S1 Fig — Each spleen was weighed and recorded to nearest gram (a), then sliced in half using sterile disposal blades (b). One half of the spleen was sliced longitudinally (c) and used for downstream experiments, while the second half was processed into paraffinised spleen tissue blocks. A subset of n = 6 Giemsa-stained spleens were read by 2 expert microscopists to validate non-phagocytosed IE counts, including the ability to distinguish into the different splenic architectural zones and parasite stages (d). Each reader examined spleen sections from different spleen biopsies in the same patient. IE, infected erythrocyte; RBC, red blood cell. (TIF) [file pmed.1003632.s001.TIF]

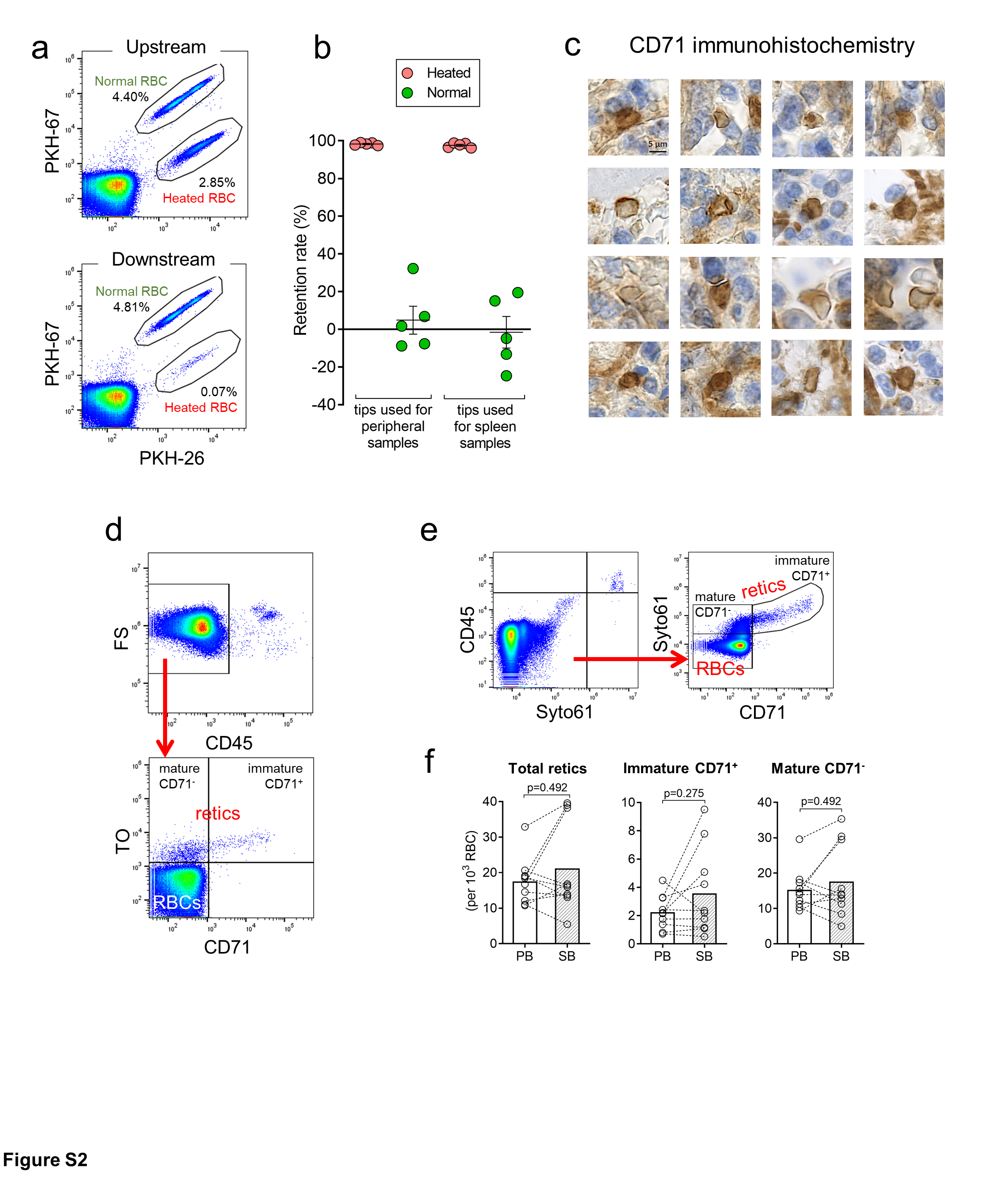

Supplement: S2 Fig — Heated and normal RBCs were used for quality control of microsphere tips and gated as shown (a). Retention rates (mean ± standard error) of >95% and <5% for heated and normal RBCs, respectively, satisfied minimum requirements (b). Immature CD71+ reticulocytes (retics) in P. falciparum- and P. vivax-infected spleens were stained by immunohistochemistry with antibodies against the CD71 transferrin receptor; additional examples of retics are shown (c). A 3-colour flow cytometry stain was used to quantitate retics in SW obtained from the flushing of normal spleens in France, identified as CD45−thiazole-orange+ cells, with or without CD71 expression (d). A 3-colour flow cytometry panel was used to phenotype retics in the Indonesian cohort, identified as CD45−SYTO61+ cells with or without CD71 (e). Numbers per thousand RBCs were determined for total, immature CD71+ and mature CD71− retics in PB and sliced SB samples (n = 10 pairs, Wilcoxon test, f). Bars represent medians. FS, forward scatter; PB, peripheral blood; RBC, red blood cell; SB, spleen blood; SW, spleen wash; TO, thiazole-orange. (TIF) [file pmed.1003632.s002.TIF]

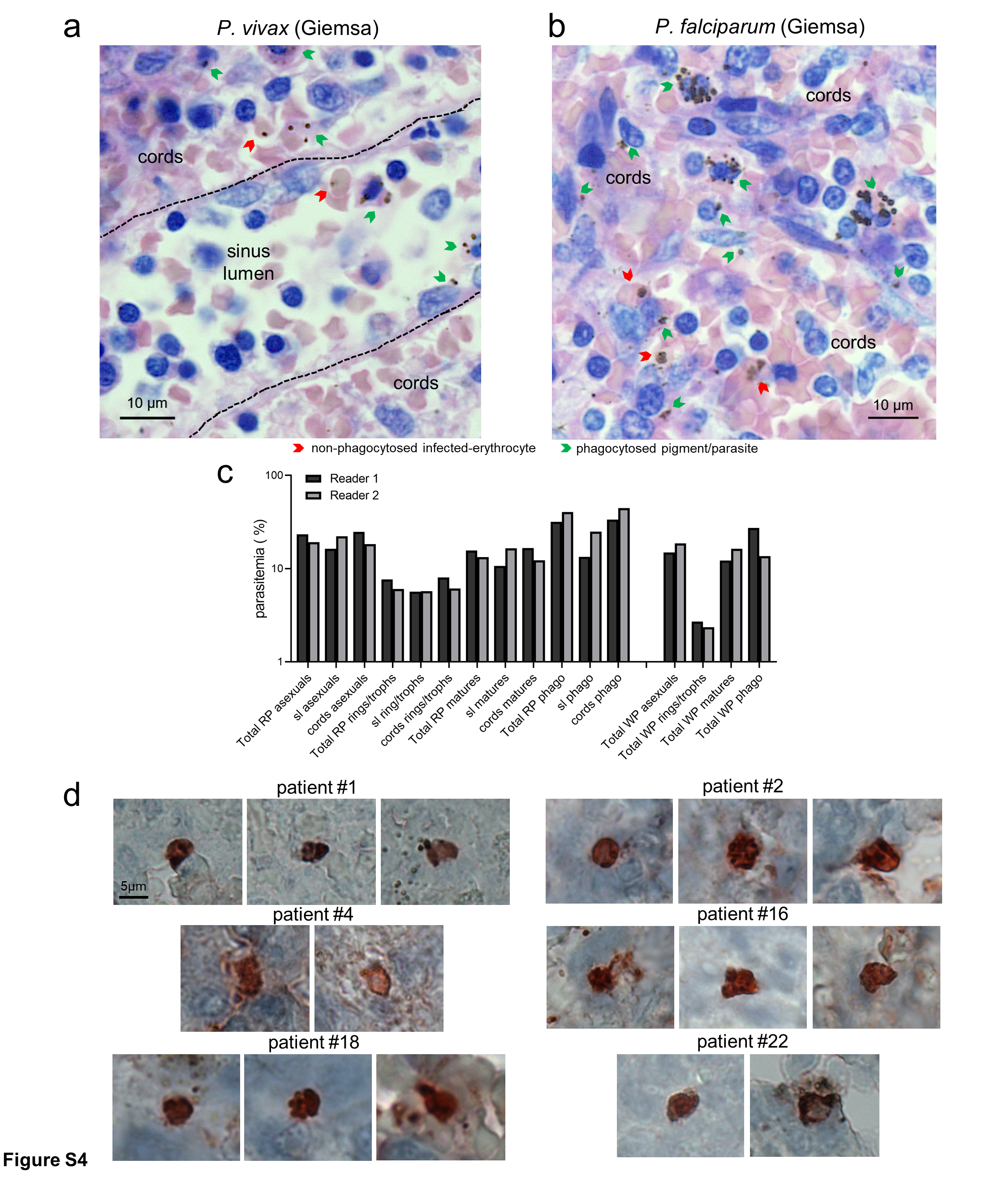

Supplement: S4 Fig — Representative images of Giemsa-stained spleen sections from P. vivax- (a, patient #1) and P. falciparum-infected spleens (b, patient #20) showing non-phagocytosed IEs (red arrowhead), pigmented phagocytes (green arrowheads) in the cords and sinus lumen. Giemsa-stained sections from the ex vivo perfused spleen (Fig 1C) were read by 2 expert microscopists (c). Reading included 5 fields of RP comprising cords and sinus lumen (sl), and 5 fields of WP, with the resulting counts in close agreement (<15% variation from the mean of readings). Additional images from immunohistochemical stains using antibodies against PvAMA1 were taken from several P. vivax-infected spleen sections showing merozoites/mature stages staining red (d). IE, infected erythrocyte; PvAMA1, Pv apical membrane antigen-1; RP, red-pulp; sl, sinus lumen; WP, white-pulp. (TIF) [file pmed.1003632.s004.TIF]
