# Supplementary material for: Evaluation of splenic accumulation and colocalization of immature reticulocytes and Plasmodium vivax in asymptomatic malaria: A prospective human splenectomy study
Source: PLoS Med. 2021 May 26;18(5):e1003632. doi: 10.1371/journal.pmed.1003632 (PMC8154101; doi:10.1371/journal.pmed.1003632)

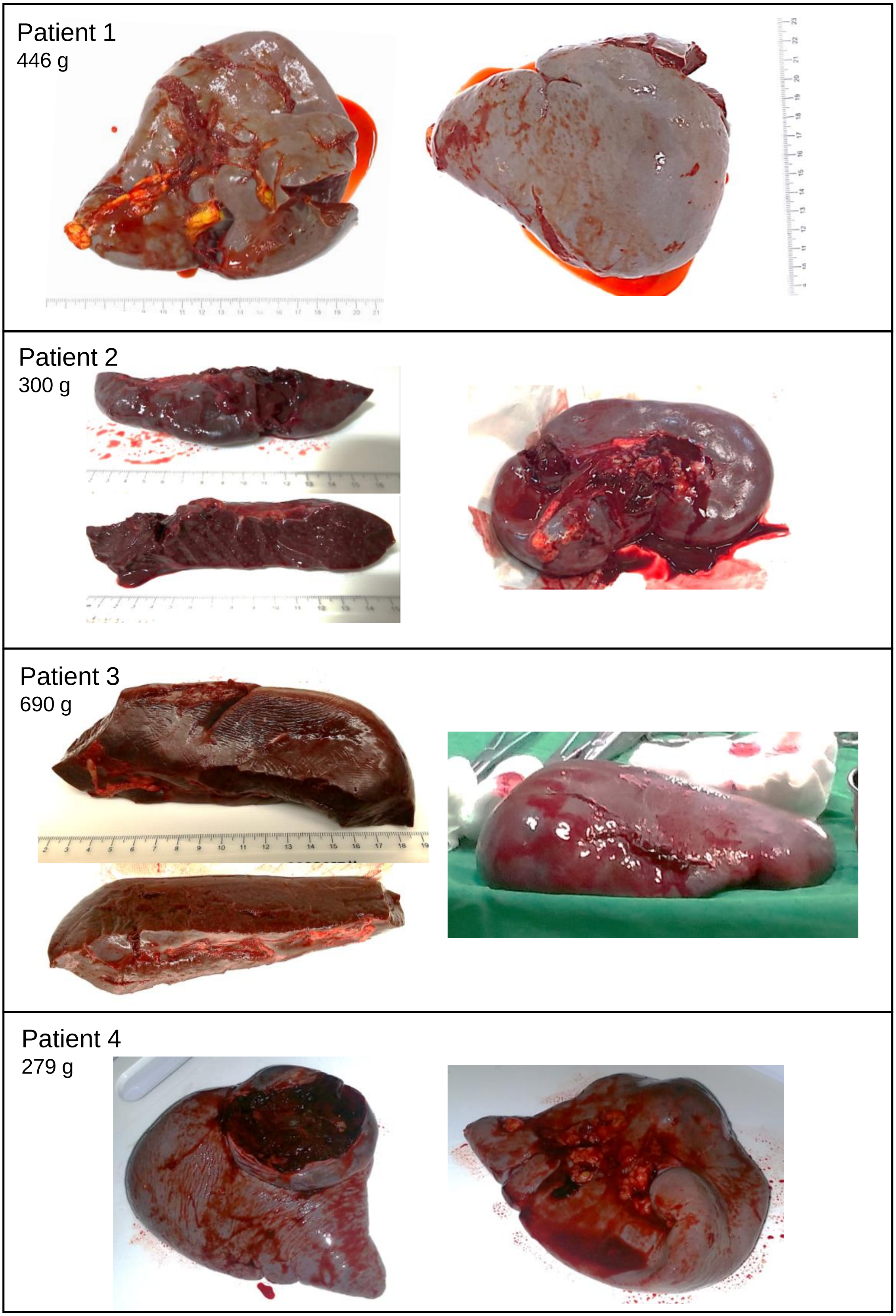
Patient 1
446 g
Patient 2
300 g
Patient 3
690 g
Patient 4
279 g

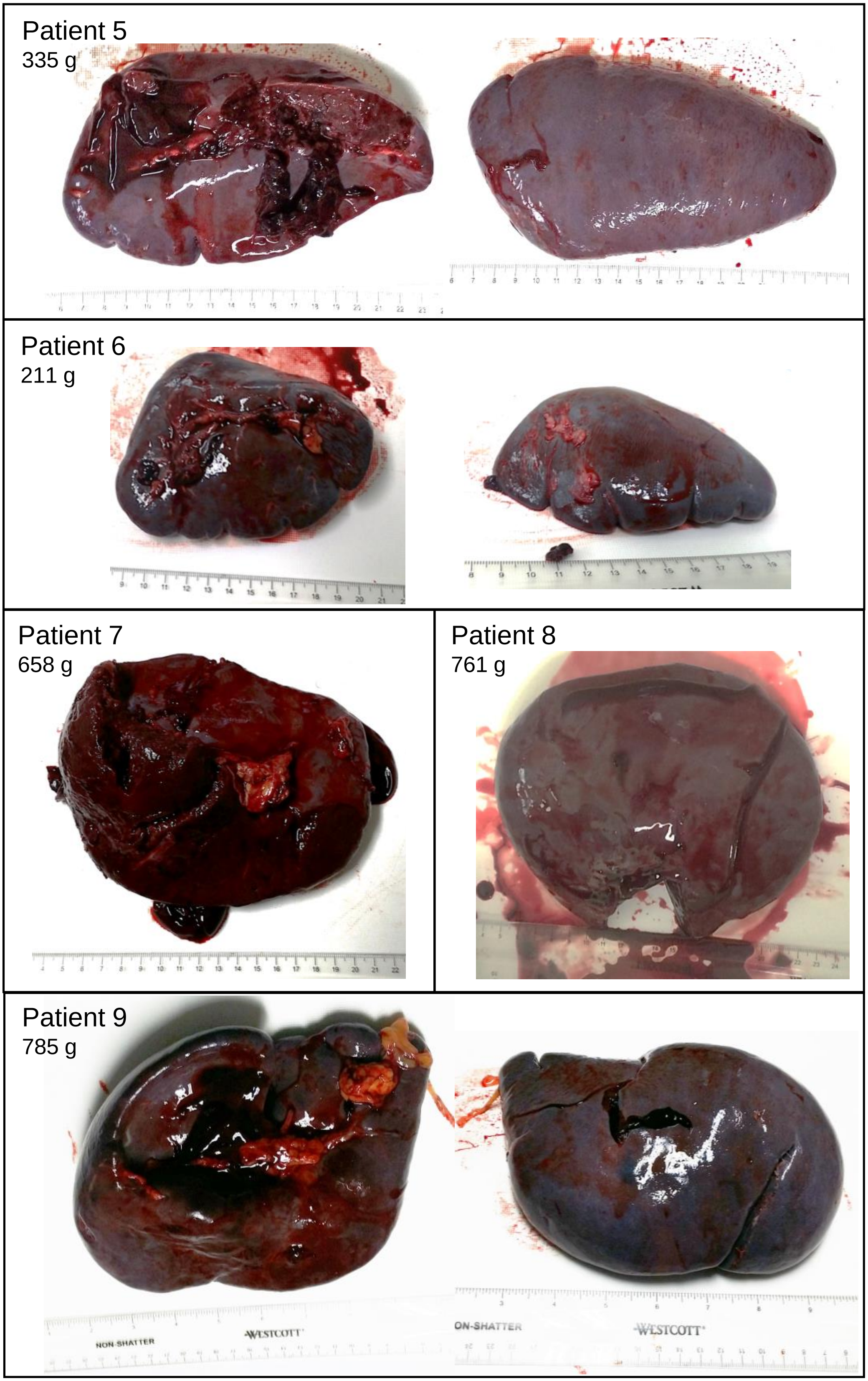
Patient 5
335 g
Patient 6
211 g
Patient 7
658 g
Patient 8
761 g
Patient 9
785 g
NON-SHATTER
WESTCOTT

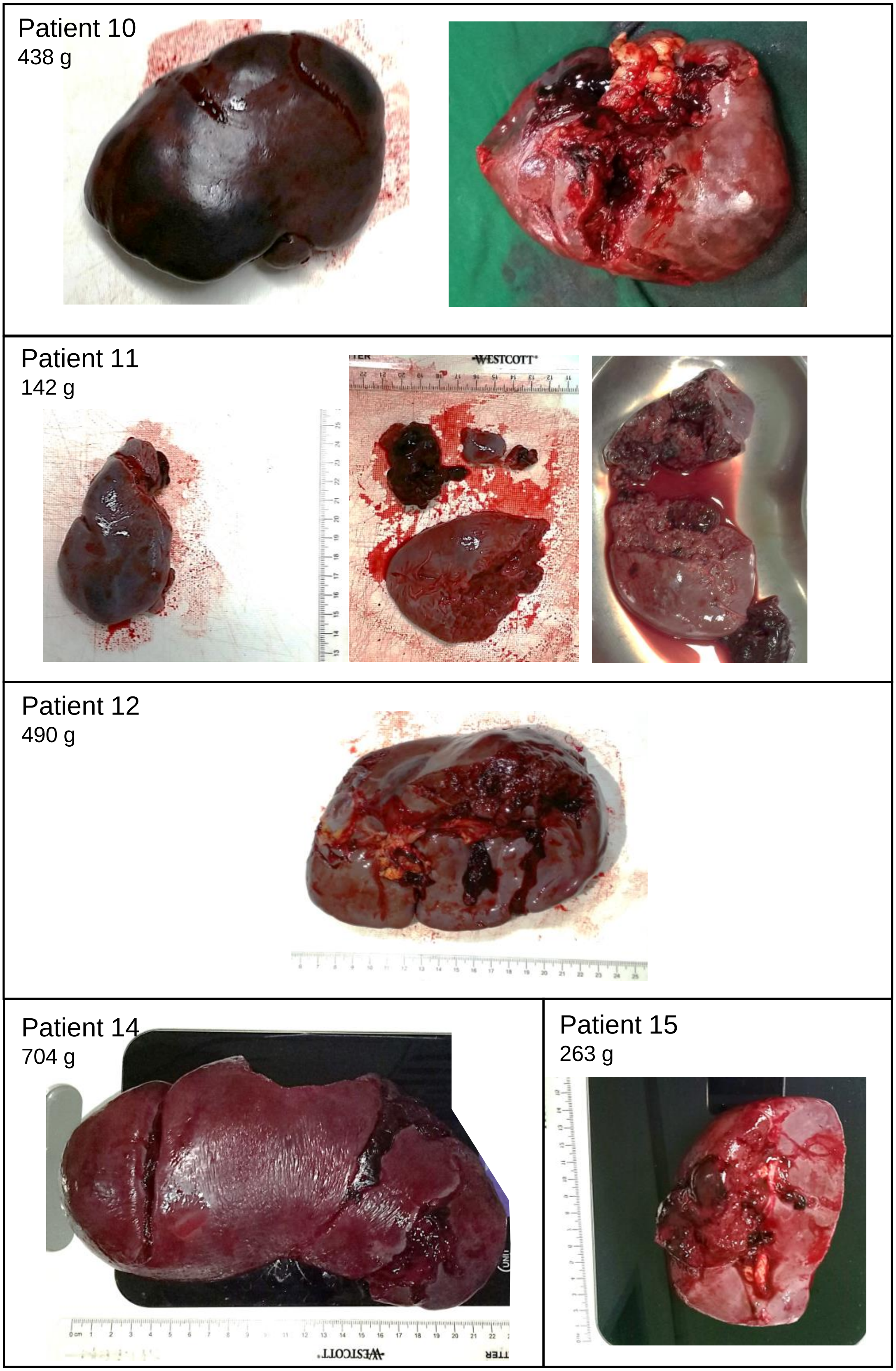
Patient 10
438 g
Patient 11
142 g
WESTCOTT
Patient 12
490 g
Patient 14
704 g
WESTCOTT
Patient 15
263 g

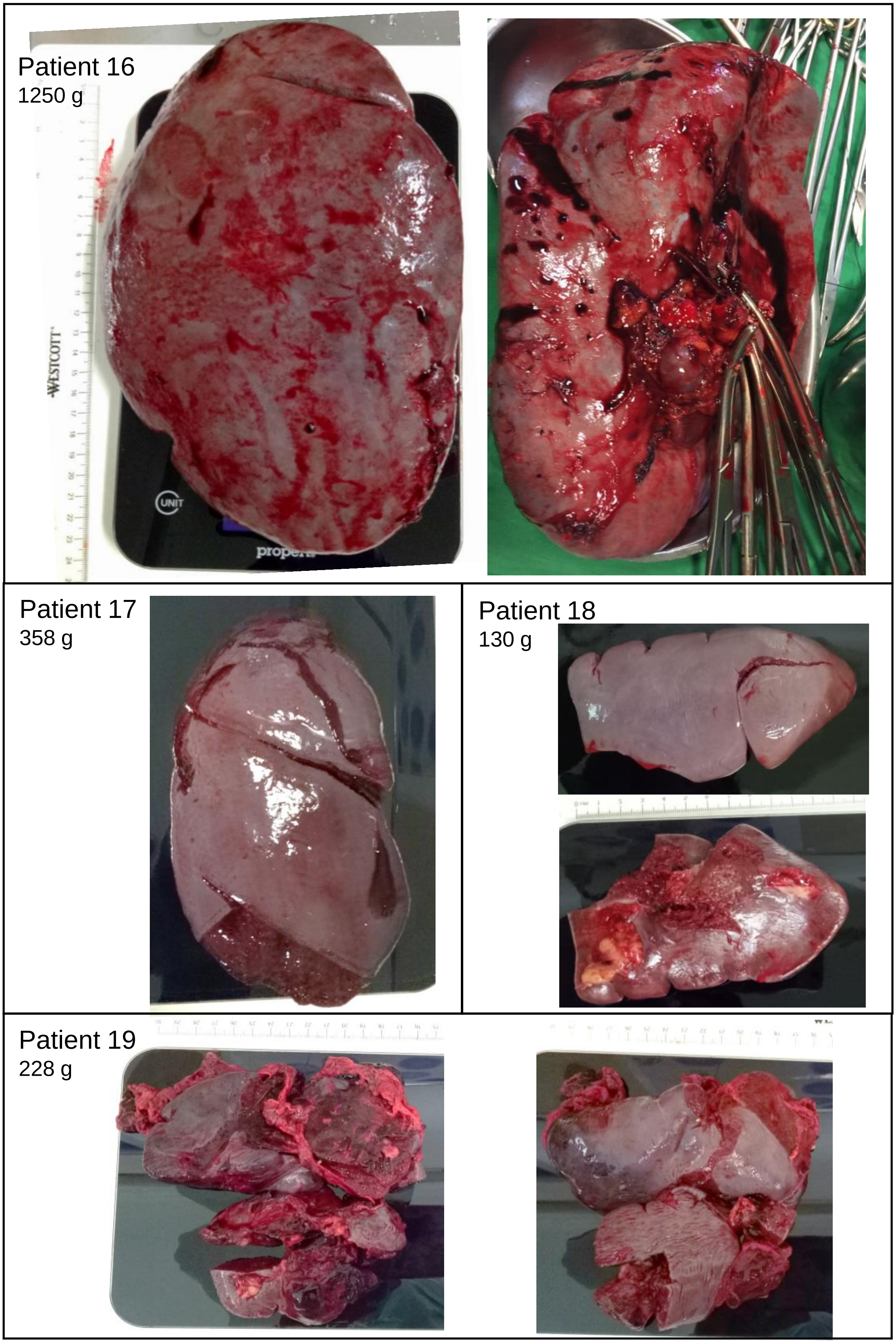
Patient 16
1250 g
Patient 17
358 g
Patient 18
130 g
Patient 19
228 g

Patient 20
1918 g

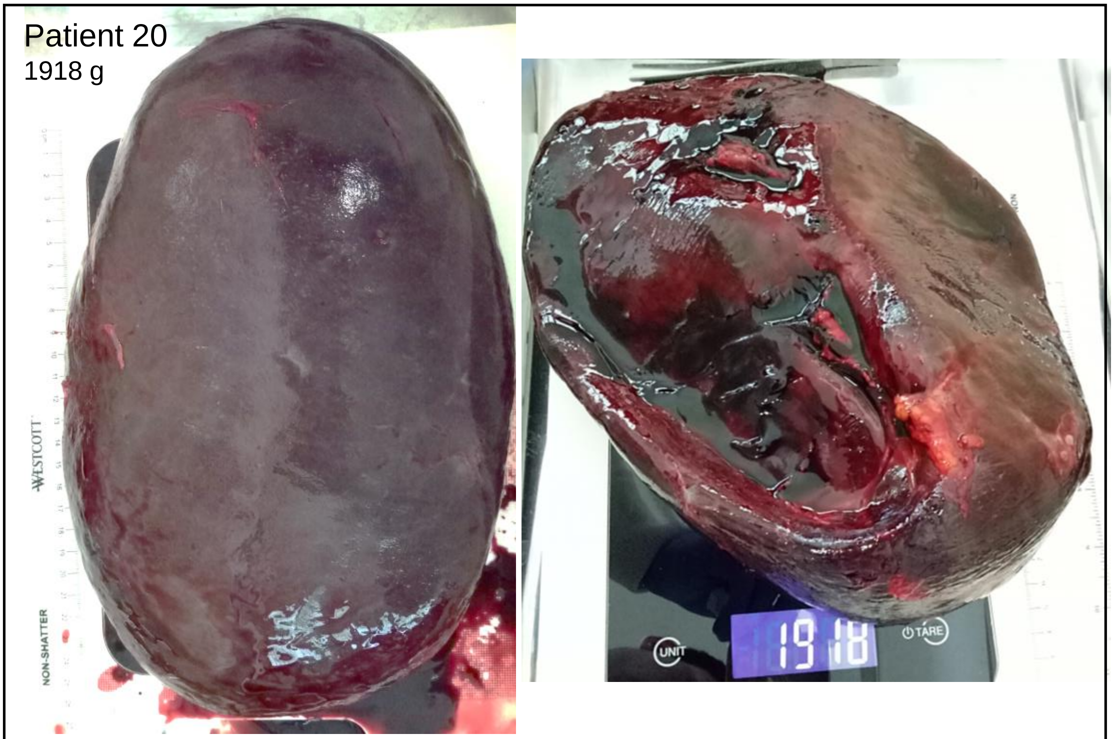

Patient 21
454 g

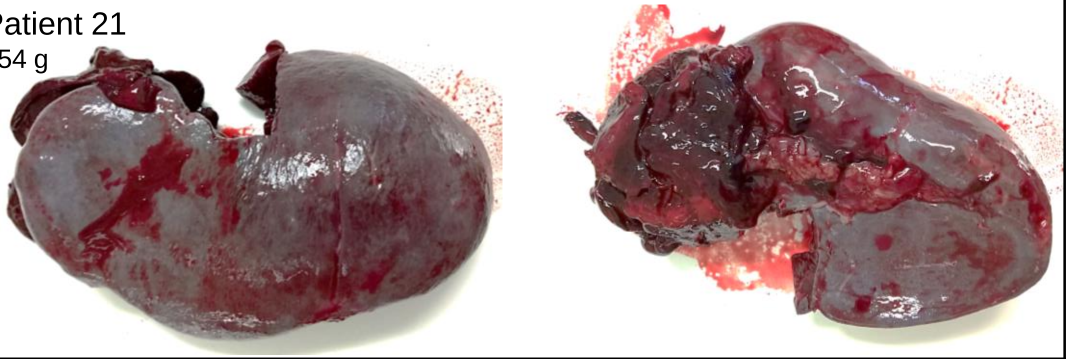

Patient 22
80 g

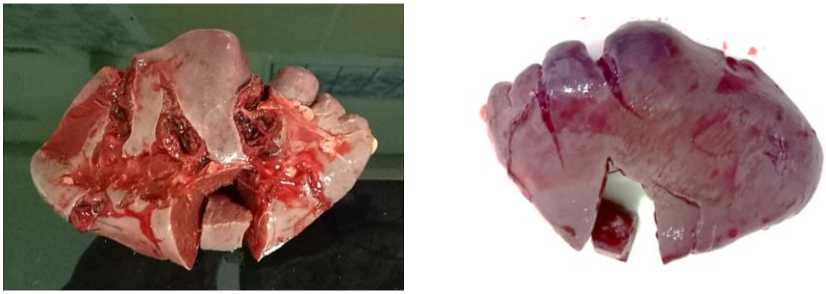

Supplement: S3 Fig — Each patient’s spleen was photographed for macroscopic records. Images not available for patient #13. Spleen weights presented in grams. (PDF) [file pmed.1003632.s003.pdf]
