# Supplementary material for: Evaluation of splenic accumulation and colocalization of immature reticulocytes and Plasmodium vivax in asymptomatic malaria: A prospective human splenectomy study
Source: PLoS Med. 2021 May 26;18(5):e1003632. doi: 10.1371/journal.pmed.1003632 (PMC8154101; doi:10.1371/journal.pmed.1003632)
Supplement: S1 Table — (DOCX) [file pmed.1003632.s005.docx]

## Table S1. *Ex-vivo* growth of peripheral and splenic *Plasmodium* parasites

| Patient  ID | Peripheral blood | | | Sliced spleen blood | | | Malaria history in last month | | |
| --- | --- | --- | --- | --- | --- | --- | --- | --- | --- |
|  | Infection | *Ex-vivo* growth | * Starting parasitaemia (%) | Infection | *Ex-vivo* growth | * Starting parasitaemia (%) | Species | Time before splenectomy | Treatment |
| 1 | Pv | N/A | N/A | Pv | N/A | N/A | - | - | - |
| 2 | sPm | N/A | N/A | Pv | N/A | N/A | - | - | - |
| 3 | sPf | N/A | N/A | Pf | N/A | N/A | - | - | - |
| 4 | sPv | N/A | N/A | sPv | no growth | ≤0.0002 | - | - | - |
| 5 | Pf | N/A | N/A | Pf | Normal | 0.2 | Pf | 3 days | DHP+PQ |
| 6 | sPv | N/A | N/A | Pv | no growth | 0.02 | Pv | <1 month | DHP+PQ |
| 7 | Pf | 24h delay | 0.003 | Pf | 24h delay | 0.04 | Pf, Pv | <1 month | DHP+PQ |
| 8 | Pf | no growth | 0.022 | Pf | no growth | ≤0.0002 | Pf | 9.5 hours | IV-ART |
| 9 | sPf | no growth | ≤0.0003 | Pf | 24h delay | 0.02 | - | - | - |
| 10 | Pf | normal | 0.018 | Pf | normal | 0.04 | - | - | - |
| 11 | neg | no growth | 0 | ^Pf | no growth | ≤0.0002 | - | - | - |
| 12 | neg | N/A | N/A | sPf | no growth | ≤0.0002 | Pf | 3 days | DHP+PQ |
| 13 | Pf | 24h delay | 0.003 | Pf | normal | 0.2 | - | - | - |
| 14 | Pf | N/A | N/A | Pf | normal | 0.1 | - | - | - |
| 15 | Pf | normal | 0.002 | Pf | 24 delay | 0.1 | - | - | - |
| 16 | sPv | no growth | ≤0.0003 | sPv | no growth | ≤0.0002 | - | - | - |
| 17 | Pf | normal | 0.16 | Pf | normal | 1.3 | - | - | - |
| 18 | sPv | no growth | ≤0.0002 | sPv | no growth | ≤0.0002 | - | - | - |
| 19 | neg | no growth | 0 | neg | no growth | 0 | - | - | - |
| 20 | neg | no growth | 0 | ^Pf | no growth | ≤0.0002 | - | - | - |
| 21 | sMix | Detectable post 48h | ≤0.0002 | sMix | Detectable post 192h | ≤0.0002 | - | - | - |
| 22 | sPv | no growth | ≤0.0002 | sPv | no growth | ≤0.0002 | - | - | - |

Footnotes:

* Samples with submicroscopic parasitaemia were assigned a starting parasitaemia value of ≤0.0003% or ≤0.0002%, calculated based on a limit of detection by microscopy of 10 parasites/µL.

^ Patient 11 and 20 were negative in the nested PCR assay but had parasites visible in spleen sections and positive *P. falciparum* signals by real time-PCR.

Splenic parasite culture experiments commenced from patient 4 onwards, and peripheral parasites from patient 7 onwards. Peripheral bloods were unavailable for patient 12 and 14.

Abbreviations: Pv, *P. vivax*; sPv, submicroscopic Pv; Pf, *P. falciparum*; sPf, submicroscopic Pf; sPm, submicroscopic *P. malariae*; sMix, submicroscopic Pf and Pv; N/A, not available; DHP, dihydroartemisinin-piperaquine; PQ, primaquine; IV-ART, intravenous artesunate.
