# Supplementary material for: Evaluation of splenic accumulation and colocalization of immature reticulocytes and Plasmodium vivax in asymptomatic malaria: A prospective human splenectomy study
Source: PLoS Med. 2021 May 26;18(5):e1003632. doi: 10.1371/journal.pmed.1003632 (PMC8154101; doi:10.1371/journal.pmed.1003632)
Supplement: S2 Table — (DOCX) [file pmed.1003632.s006.docx]

## Table S2. Baseline characteristics of patients with reticulocytes evaluated by flow cytometry

| Patient ID | Age | Gender ^a^ | Ethnicity ^b^ | Reason for  splenectomy | Body  Temp  (°C) | Spleen  weight  (grams) ^c^ | *Plasmodium* infection | FU month | RBC count  (x10^6^/µL) | | Haemoglobin  (g/dL) | | Platelet count  (x10^3^/µL) | |
| --- | --- | --- | --- | --- | --- | --- | --- | --- | --- | --- | --- | --- | --- | --- |
|  |  |  |  |  |  |  |  |  | Surgery | FU | Surgery | FU | Surgery | FU |
| 1 | 36 | M | H | trauma | 36.5 | 446 (S) | *P. vivax* | 4 | 4.92 | 5.79 | 13.7 | 12.6 | 153 | 645 |
| 2 | 24 | F | NP | trauma | 36.5 | 300 (S) | *P. vivax* | 5 | 4.10 | 4.85 | 9.3 | 9.9 | 147 | 463 |
| 3 | 16 | M | H | trauma | N/A | 690 (S) | *P. falciparum* | 3 | 5.00 | 5.47 | 11.6 | 11.9 | 189 | 515 |
| 4 | 46 | M | L | trauma | 37.0 | 279 (S) | *P. vivax* | 2 | 2.79 | 4.29 | 7.4 | 11.7 | 251 | 155 |
| 5 | 15 | M | L | trauma | 36.2 | 335 (S) | *P. falciparum* | 2 | 3.59 | 5.85 | 7.2 | 11.4 | 72 | 199 |
| 6 | 35 | M | H | trauma | 36.0 | 211 (N) | *P. vivax* | 2 | 4.97 | 5.09 | 15.1 | 14.3 | 266 | 544 |
| 7 | 20 | M | H | trauma | 36.3 | 658 (S) | *P. falciparum* | 3 | 3.36 | 5.03 | 9.3 | 10.4 | 90 | 323 |
| 9 | 41 | M | H | trauma | 36.8 | 785 (S) | *P. falciparum* | 3 | 3.76 | 5.12 | 10.7 | 13.2 | 125 | 317 |
| 10 | 30 | M | NP | trauma | 36.8 | 438 (S) | *P. falciparum* | 2 | 4.81 | 5.20 | 13.1 | 13.7 | 222 | 367 |
| 11 | 39 | M | NP | trauma | 36.4 | 142 (N) | *P. falciparum* | 2 | 3.81 | 5.49 | 10.8 | 15.2 | 126 | 441 |
|  |  |  |  |  | Median [interquartile range] | | | | 3.96  [3.53-4.93] | 5.16  [4.99-5.57] | 10.8  [8.8-13.3] | 12.3  [11.2-13.9] | 150  [116-229] | 404  [288-522] |
|  |  |  |  |  | Wilcoxon test *P*-value | | | | 0.002 | | 0.070 | | 0.004 | |

Footnotes:

^a^ M, male; F, female.

^b^ H, highland Papuan; L, lowland Papuan; NP, non-Papuan.

^c^ N, normal (<250 g); S, splenomegaly (250-1,000 g); SS, severe splenomegaly (>1,000 g).

None of the patients were seropositive for the human immunodeficiency virus, fever (≥37.5°C) or malaria symptoms at surgery.

Reticulocytes were examined fresh by flow cytometry for the first 11 patients in the cohort (except patient 8). Experiments ceased thereafter due to technical difficulties at the field facility.

Abbreviations: N/A, not available (missing); FU, follow-up; RBC, red blood cell.
