# Supplementary material for: Evaluation of splenic accumulation and colocalization of immature reticulocytes and Plasmodium vivax in asymptomatic malaria: A prospective human splenectomy study
Source: PLoS Med. 2021 May 26;18(5):e1003632. doi: 10.1371/journal.pmed.1003632 (PMC8154101; doi:10.1371/journal.pmed.1003632)
Supplement: S3 Table — (DOCX) [file pmed.1003632.s007.docx]

## Table S3. Baseline characteristics of n=9 spleno-pancreatectomy patients in France

| Patient  ID | Age  (years) | Reason for splenectomy | | Spleen weight  (grams) |
| --- | --- | --- | --- | --- |
|  |  |  |  |  |
| 1 | 64 | adenocarcinoma | | 150 |
| 2 | 78 | adenocarcinoma | | 198 |
| 3 | 68 | adenocarcinoma | | 70 |
| 4 | 44 | intrapapillary pancreatic tumor | | 70 |
| 5 | 65 | adenocarcinoma | | 200 |
| 6 | 52 | neuroendocrine tumor in the pancreas | | 170 |
| 7 | 55 | Wirsung rupture due to chronic pancreatitis | | 220 |
| 8 | 85 | pancreatic cyst | | 70 |
| 9 | 53 | adenocarcinoma | | 80 |
|  |  |  |  |  |
|  |  |  |  |  |
