# Supplementary material for: Evaluation of splenic accumulation and colocalization of immature reticulocytes and Plasmodium vivax in asymptomatic malaria: A prospective human splenectomy study
Source: PLoS Med. 2021 May 26;18(5):e1003632. doi: 10.1371/journal.pmed.1003632 (PMC8154101; doi:10.1371/journal.pmed.1003632)
Supplement: S4 Table — (DOCX) [file pmed.1003632.s008.docx]

## Table S4. Full blood counts at surgery

##

| Patient  ID | Infection ^a^ | Spleen  Size ^b^ | White blood cells (counts x10^3^ per µL) | | | | | | Red blood cells | | | | | | | Platelets | | | |
| --- | --- | --- | --- | --- | --- | --- | --- | --- | --- | --- | --- | --- | --- | --- | --- | --- | --- | --- | --- |
|  |  |  | total | neutro | lymph | mono | eos | baso | count  (x10^6^ per µL) | Hb (g/dL) | Hct (%) | MCV (fL) | MCH (pg) | RDW-CV (%) | RDW-SD (fL) | count (x10^3^ per µL) | PDW (fL) | P-LCR (%) | MPV (fL) |
| 1 | Pv | S | 9.54 | 5.16 | 2.58 | 0.60 | 1.19 | 0.01 | 4.92 | 13.7 | 38.4 | 78.0 | 27.8 | 13.8 | 37.7 | 153 | 11.5 | 25.6 | 10.2 |
| 2 | Pv | S | 14.86 | N/A | N/A | N/A | N/A | N/A | 4.10 | 9.3 | 29.1 | 71.0 | 22.7 | 14.5 | 35.9 | 147 | 11.3 | 27.2 | 10.4 |
| 3 | Pf | S | 11.29 | 8.56 | 1.89 | 0.60 | 0.25 | 0 | 5.00 | 11.6 | 34.2 | 68.4 | 23.2 | 15.3 | 37.3 | 189 | 12.2 | 27.2 | 10.0 |
| 4 | Pv | S | 7.28 | 4.24 | 1.37 | 0.68 | 0.96 | 0.03 | 2.79 | 7.4 | 21.9 | 78.5 | 26.5 | 12.4 | 33.7 | 251 | 7.9 | 10.1 | 8.1 |
| 5 | Pf | S | 4.20 | N/A | N/A | N/A | N/A | N/A | 3.59 | 7.2 | 22.5 | 62.7 | 20.1 | 16.0 | 34.9 | 72 | N/A | N/A | N/A |
| 6 | Pv | N | 10.46 | 4.30 | 4.93 | 0.28 | 0.91 | 0.04 | 4.97 | 15.1 | 41.5 | 83.5 | 30.4 | 12.9 | 38.2 | 266 | 10.8 | 21.9 | 9.6 |
| 7 | Pf | S | 6.40 | 4.12 | 1.51 | 0.47 | 0.27 | 0.03 | 3.36 | 9.3 | 27.3 | 81.3 | 27.7 | 14.1 | 38.4 | 90 | 11.0 | 23.2 | 9.7 |
| 8 | Pf | S | 18.63 | 14.31 | 3.37 | 0.93 | 0.02 | 0 | 3.38 | 10.2 | 28.8 | 85.2 | 30.2 | 13.5 | 39.2 | 276 | 9.8 | 20.2 | 9.4 |
| 9 | Pf | S | 13.41 | 10.88 | 1.74 | 0.72 | 0.05 | 0.01 | 3.76 | 10.7 | 32.6 | 86.7 | 28.5 | 14.8 | 45.5 | 125 | 15.3 | 40.6 | 12.0 |
| 10 | Pf | S | 17.24 | 14.36 | 1.71 | 1.14 | 0.02 | 0.02 | 4.81 | 13.1 | 38.1 | 79.2 | 27.2 | 13.3 | 37.2 | 222 | 11.4 | 25.5 | 9.9 |
| 11 | Pf | N | 19.46 | 17.84 | 0.95 | 0.62 | 0.02 | 0.02 | 3.81 | 10.8 | 31.2 | 81.9 | 28.3 | 12.2 | 35.0 | 126 | 12.2 | 29.2 | 10.6 |
| 12 | Pf | S | 7.92 | 4.78 | 1.54 | 0.62 | 0.96 | 0.02 | 2.85 | 6.1 | 21.9 | 76.8 | 21.4 | 20.1 | 53.2 | 263 | 14.7 | 35.8 | 11.3 |
| 13 | Pf | S | 9.59 | 4.05 | 4.01 | 0.74 | 0.79 | 0.01 | 2.85 | 6.7 | 23.3 | 81.8 | 23.5 | 27.6 | 73.0 | 200 | 9.7 | 18.4 | 9.2 |
| 14 | Pf | S | 4.70 | 2.11 | 1.66 | 0.68 | 0.23 | 0.02 | 3.00 | 6.3 | 20.8 | 69.3 | 21.0 | 22.8 | 46.6 | 144 | 16.1 | 40.6 | 11.6 |
| 15 | Pf | S | 13.15 | 7.93 | 4.01 | 0.95 | 0.22 | 0.04 | 3.48 | 10.5 | 30.9 | 88.8 | 30.2 | 12.9 | 39.9 | 149 | 10.5 | 23.3 | 9.9 |
| 16 | Pv | SS | 2.17 | N/A | N/A | N/A | N/A | N/A | 3.74 | 9.5 | 29.7 | 79.4 | 25.4 | 16.0 | 44.9 | 70 | N/A | N/A | N/A |
| 17 | Pf | S | 8.87 | N/A | N/A | N/A | N/A | N/A | 3.40 | 10.9 | 32.0 | 94.1 | 32.1 | 19.1 | 47.6 | 134 | 11.3 | 31.6 | 10.9 |
| 18 | Pv | N | 18.72 | 10.91 | 6.68 | 0.39 | 0.69 | 0.04 | 4.92 | 12.4 | 39.3 | 79.9 | 25.2 | 12.6 | 35.5 | 276 | 10.6 | 26.7 | 10.4 |
| 19 | neg | N | 28.48 | N/A | N/A | N/A | N/A | N/A | 3.27 | 9.7 | 28.9 | 88.4 | 29.7 | 12.5 | 38.8 | 144 | 9.8 | 21.6 | 9.8 |
| 20 | Pf | SS | 2.49 | 1.54 | 0.74 | 0.12 | 0.08 | 0.01 | 3.02 | 7.1 | 23.0 | 76.2 | 23.5 | 17.6 | 46.3 | 37 | 11.5 | 21.0 | 9.2 |
| 21 | Mix | S | 13.66 | 8.50 | 2.93 | 1.10 | 1.11 | 0.02 | 4.28 | 12.5 | 36.1 | 84.3 | 29.2 | 17.8 | 52.0 | 180 | 9.9 | 21.3 | 9.6 |
| 22 | Pv | N | 16.11 | 7.69 | 7.52 | 0.59 | 0.27 | 0.04 | 4.19 | 10.8 | 33.6 | 80.2 | 25.8 | 13.2 | 38.0 | 266 | 14.8 | 42.7 | 12.3 |

Footnotes:

^a^ Pv, *P. vivax*; Pf, *P. falciparum*; Mix, Pf and Pv; ND, not detected.

^b^ N, normal (<250 g); S, splenomegaly (250-1,000 g); SS, severe splenomegaly (>1,000 g).

White cell differentials unavailable for five patients due to analyser limitations.

Abbreviations: N/A, not available (missing); Hb, haemoglobin; Hct, haematocrit; MCV, mean corpuscular volume; MCH, mean corpuscular haemoglobin; RDW-CV, red blood cell distribution width – coefficient of variation; RDW-SD, RDW – standard deviation; PDW, platelet distribution width; P-LCR, platelet large cell ratio; MPV, mean platelet volume
